# Genomic Drivers of Coronary Artery Disease and Risk of Future Outcomes After Coronary Angiography

**DOI:** 10.1001/jamanetworkopen.2024.55368

**Published:** 2025-01-21

**Authors:** Kelvin Supriami, Sarah M. Urbut, José R. Tello-Ayala, Ozan Unlu, Samuel F. Friedman, Roukoz Abou-Karam, Satoshi Koyama, Md Mesbah Uddin, Eugene Pomerantsev, Michael T. Lu, Michael C. Honigberg, Krishna G. Aragam, Finale Doshi-Velez, Aniruddh P. Patel, Pradeep Natarajan, Patrick T. Ellinor, Akl C. Fahed

**Affiliations:** 1 Department of Medicine, Harvard Medical School, Boston, Massachusetts; 2 Cardiovascular Disease Initiative, Broad Institute of MIT and Harvard, Cambridge, Massachusetts; 3 Division of Cardiology, Massachusetts General Hospital, Boston; 4 John A. Paulson School of Engineering and Applied Sciences, Harvard University, Cambridge, Massachusetts; 5 Division of Cardiology, Brigham and Women’s Hospital, Boston, Massachusetts; 6 Department of Biomedical Informatics, Harvard Medical School, Boston, Massachusetts; 7 Cardiovascular Imaging Research Center, Department of Radiology, Massachusetts General Hospital & Harvard Medical School, Boston; 8 Center for Genomic Medicine, Massachusetts General Hospital, Boston; 9 Personalized Medicine, Mass General Brigham, Boston, Massachusetts

## Abstract

**Importance:**

Disease characteristics of genetically mediated coronary artery disease (CAD) on coronary angiography and the association of genomic risk with outcomes after coronary angiography are not well understood.

**Objective:**

To assess the angiographic characteristics and risk of post–coronary angiography outcomes of patients with genomic drivers of CAD: familial hypercholesterolemia (FH), high polygenic risk score (PRS), and clonal hematopoiesis of indeterminate potential (CHIP).

**Design, Setting, and Participants:**

A retrospective cohort study of 3518 Mass General Brigham Biobank participants with genomic information who underwent coronary angiography was conducted between July 18, 2000, and August 1, 2023.

**Exposures:**

The presence of a genomic risk factor of CAD, defined as FH variant, high CAD PRS, or CHIP driver variation.

**Main Outcomes and Measures:**

Coronary artery disease presentation (stable or acute), angiographic CAD characteristics (severity and burden), angiographic outcomes (repeat angiogram, revascularization, and in-stent restenosis), and clinical outcomes (heart failure and all-cause mortality).

**Results:**

Among 3518 participants (2467 [70.1%] male; median age, 64.0 [IQR, 55.0-72.0] years), 1509 (42.9%) had at least 1 genomic driver of CAD (26 FH, 1191 high CAD PRS, and 466 CHIP) that was associated with the presentation of acute coronary syndromes (adjusted odds ratio, 2.67; 95% CI, 2.19-3.26) and with the presence, burden, and severity of angiographic CAD. This association was driven by FH and CAD PRS. One SD of CAD PRS was associated with a 12.51-point higher Gensini score. During 9 years of follow-up, there was an increased risk among FH carriers for a repeat angiogram (adjusted hazard ratio [AHR], 1.70; 95% CI, 1.02-2.83), and revascularization (AHR, 1.97; 95% CI, 1.02-3.80), and among people with high CAD PRS (repeat angiogram: AHR, 1.79; 95% CI, 1.45-2.22; revascularization: AHR, 1.85; 95% CI, 1.37-2.50; and in-stent restenosis: AHR, 3.89; 95% CI, 2.16-7.01). CHIP carriers had no significant increase in angiographic outcomes but were at higher risk of heart failure (AHR, 1.58; 95% CI, 1.04-2.40) and all-cause mortality (AHR, 1.78; 95% CI, 1.47-2.16).

**Conclusions and Relevance:**

The findings of this study suggest that germline monogenic and polygenic risk are associated with acute coronary syndromes presentation, severity and burden of atherosclerosis, and risk of repeat angiogram, revascularization, and in-stent restenosis. CHIP variant status is associated with incident heart failure and mortality after coronary angiography.

## Key Points

**Question:**

Are genomic drivers of coronary artery disease (CAD) associated with disease severity and future outcomes in patients undergoing coronary angiography?

**Findings:**

In a cohort study of 3518 participants undergoing coronary angiography, familial hypercholesterolemia variants and polygenic risk score, but not clonal hematopoiesis of indeterminate potential (CHIP), were associated with angiographic severity and burden. Familial hypercholesterolemia carrier status and CAD polygenic risk score were associated with the risk of repeat angiogram, future revascularization, and in-stent restenosis; CHIP was associated with future heart failure and all-cause mortality risk.

**Meaning:**

The findings of this study suggest that genomic drivers of CAD are associated with the atherosclerosis burden observed angiographically and portend the risk of adverse outcomes.

## Teaser

This cohort study examines angiographic outcomes of patients with familial hypercholesterolemia, high polygenic risk score, and clonal hematopoiesis of indeterminate potential as genomic drivers of coronary artery disease.

## Introduction

Integrating genomic information with clinical risk factors augments coronary artery disease (CAD) risk prediction, a disease with 40% to 60% heritability.^1,2,3,4,5^ Key genomic drivers of CAD risk comprise germline and somatic variations. Familial hypercholesterolemia (FH), a tier 1 monogenic disease characterized by lifelong increased low-density lipoprotein cholesterol levels, affects 1 in 250 patients.^6,7,8,9^ Familial hypercholesterolemia variant carriers have a 2- to 3-fold increased risk of early-onset CAD.^6,10^ A CAD polygenic risk score (PRS) aggregates the small effects of common DNA variants into a single quantitative predictor. Individuals with high CAD PRS (typically the top 20% of the population distribution of CAD PRS) carry a CAD risk comparable to that of FH carriers.^4,10,11,12^ In addition to germline risk, clonal hematopoiesis of indeterminate potential (CHIP) due to age-related somatic variants is associated with a 1.4-fold increased CAD risk.^13,14^

While associations of these genomic drivers with first incident CAD events are well established in large cohorts, little is known about how genetically driven disease might present on coronary imaging among patients with known or suspected CAD. Coronary angiography is the highest standard imaging modality for evaluating CAD and allows for percutaneous coronary intervention during the same procedure.^15^ Coronary angiography provides detailed anatomic characterization of coronary atherosclerosis, such as plaque location, severity, and burden, which is highly prognostic for recurrent cardiovascular events and mortality and often guides revascularization decisions.^16^

While genomics is most useful for primary prevention and early risk stratification, recent work indicates a potential in secondary prevention.^17^ Individuals undergoing coronary angiography often have high pretest probability due to prior CAD symptoms and/or testing. It is uncertain whether genomic information adds estimation value for future events in these patients. To our knowledge, no study has yet incorporated these 3 genomic drivers to understand CAD heterogeneity on imaging and assess comprehensive outcomes.

Herein, we report on a cohort study of patients with genomic data who underwent coronary angiography, comparing CAD presentation and characterization across different genomic drivers and against patients without genomic drivers. We then examine whether genomic risk is associated with future angiographic and clinical outcomes in patients undergoing coronary angiography.

## Methods

### Study Population

Participants in the Mass General Brigham Biobank who underwent their first coronary angiography and had available genomic data were included (N = 3518). Mass General Brigham Biobank is a hospital-based biobank that currently includes 53 125 participants with genomic data.^18^ Coronary angiography information was derived from a structured database of cardiac catheterization performed between July 18, 2000, and August 1, 2023. Participants were followed up through October 20, 2023, for incident outcomes (eFigure 1 in Supplement 1). This study was approved by the Mass General Brigham Institutional Review Board, with requirement for informed consent waived, and followed the Strengthening the Reporting of Observational Studies in Epidemiology (STROBE) reporting guideline.

### Ascertainment of Genomic Drivers

Carriers of a pathogenic or likely pathogenic FH variant were identified using data from whole-exome sequencing. Variants in any of 3 FH causal genes (*APOB*, *PCSK9*, and *LDLR*) were considered as previously described.^19,20^ The CAD PRS was computed using a recently validated multiancestry PRS (GPS_Mult_; accession ID, PGS003725), which consists of 1 296 172 variants.^11^ The CAD PRS was adjusted for the first 20 principal components of genetic ancestry, standardized, and categorized into 3 risk groups: low (bottom quintile), intermediate (middle 3 quintiles), and high (top quintile), as previously described (eMethods in Supplement 1).^21^ Individuals in the top quintile were considered to have a high CAD PRS based on a 3-fold increased risk for CAD compared with the population average.^11^ CHIP status was established using the same whole-exome sequencing data with at least 1 detected putative variant in 69 CHIP-driver genes, including *DNMT3A*, *TET2*, *ASXL1*, and *JAK2*, at a variant allele frequency greater than or equal to 2%.^22,23,24^ Given the lifelong dynamics of CHIP,^25,26,27^ its analyses were restricted to participants with blood samples collected within a range of 10 years before and 1 year after their initial coronary angiography.

Of all participants, 85 were excluded from FH analyses due to unavailable whole-exome sequencing data, and 1279 individuals were excluded from CHIP analyses due to ascertainment dates that were missing (n = 40) or beyond the specified range (n = 1239) (eFigure 2 in Supplement 1).

### Ascertainment of Coronary Angiographic Characteristics

The severity of CAD, defined as the most severe stenosis occurring in any coronary artery, was classified as none, mild (<50%), moderate (50%-69%), and severe (≥70%, except for left main ≥50%). We also calculated the number of coronary arteries with severe stenosis, defined as the burden of CAD, and categorized as nonobstructive, single-vessel, multivessel (2 or 3 vessels), and left main disease. Continuous measure of angiographic burden of CAD was assessed using the modified Gensini score.^28^ In brief, the Gensini score quantifies every coronary artery lesion based on stenosis degree and multiplies by a factor according to its location. The final score is a sum of all lesion severity scores. The original Gensini score includes an adjustment factor for the presence of collateral circulation in case of chronic total occlusion, which we did not include in the modified version. Initial presentations of CAD were obtained from medical record review to identify reasons for coronary angiography and were classified as stable CAD vs acute coronary syndromes (ACS).

### Ascertainment of Outcomes

Repeat angiogram was determined based on subsequent records of coronary angiograms. Revascularization was defined as the composite of coronary artery bypass grafting and percutaneous coronary intervention, excluding individuals whose revascularization occurred within 3 months after the initial angiogram to avoid bias from planned procedures. In-stent restenosis was described based on coronary angiogram reports interpreted by interventional cardiologists. Heart failure was ascertained as a confirmed diagnosis of chronic heart failure with any ejection fraction and obtained using a natural language processing system extracted from electronic health records as described previously.^29^ Mortality was defined as all-cause mortality. Occurrences and dates of death were obtained from the monthly death master file provided by the US Social Security Administration. Blocking window periods were applied for mortality (1 month) and heart failure (3-month) analyses.

### Statistical Analysis

Baseline characteristics of participants are presented as median (IQR) or mean (SD) for continuous variables based on the distribution and number with frequency for categorical variables. Associations between coronary angiography characteristics and genomic drivers of CAD were assessed using multivariable logistic or linear regression, adjusted for age at the time of coronary angiography, sex, and the first 4 principal components of genetic ancestry. The traditional cardiovascular clinical risk factors were further added as covariates in additional models of analysis (eMethods in Supplement 1). We evaluated the association of categorical characteristics with multinomial logistic regression, estimated by multinom function from the R nnet package (R Foundation for Statistical Computing).^30^

We fit Cox proportional hazards regression models for repeat angiogram, revascularization, in-stent restenosis, heart failure, and mortality with time-to-event defined as years from the first coronary angiography and with the same preselected covariates as in the previous paragraph. We considered CHIP as a time-dependent covariate in addressing dynamic risk changes before and after CHIP acquisition.^31^ In brief, for each patient, we divided their observed interval into times before and after CHIP collection if CHIP data were collected. During these intervals, the covariate for CHIP was set to either 0 (period before CHIP was detected) or 1 (period after CHIP was detected) for each patient. The cumulative incidence of an event by time *t* was estimated by F(*t*) = 1 − S(*t*) using the survfit function from the R survival package.^32^ The cumulative incidence plots were visualized using the adjusted curves R package.^33^

Three sensitivity analyses were performed. First, we conducted age- and sex-matched analyses of FH variant carriers and controls without any genomic driver to balance the distribution of covariates given the small sample size of FH. Second, analysis on mortality risk was restricted to individuals who were sequenced either before or within a maximum range of 1 year after their first coronary angiography was done to account for immortal time. Third, we investigated whether the association of CHIP is different across driver genes and clone size through 3 models: top driver genes (*DNMT3A*, *TET2*, *ASXL1*, or *JAK2*), variant allele frequency greater than or equal to 10%, and non-*DNMT3A* genes; all were compared with no CHIP. Additionally, we validated the robustness of our findings for CAD PRS in an independent replication cohort with available genotyping data from the Mass General Brigham Biobank.

Statistical analyses were performed using R, version 4.1.2. Statistical significance was set as *P* < .05, and 2-sided *P* values were used.

## Results

### Angiographic CAD

Among 3518 participants, 1051 were female (29.9%), 2467 male (70.1%), 3152 were White (89.6%); race and ethnicity designations for 366 participants were Asian 38 (1.1%), Black 135 (3.8%), other 120 (3.4%), and 73 (2.1%) did not have race reported. The median age was 64.0 (IQR, 55.0-72.0) years, 2568 (73.0%) had angiographic evidence of CAD, consisting of 340 (9.7%) with mild, 346 (9.8%) with moderate, and 1882 (53.5%) with severe stenosis. Of those with CAD, 966 individuals (27.5%) presented with ACS and 1647 (46.8%) with stable CAD, and 900 (35.0%) had 1-vessel disease, 465 (18.1%) had 2-vessel disease, 283 (11.0%) had 3-vessel disease, and 234 (9.2%) had left main disease. The median modified Gensini score was 16.0 (IQR, 0-51.0). Baseline participant characteristics are listed in the Table and eTables 1-6 in Supplement 1.

**Table.** Baseline Characteristics and Outcomes by Genomic Risk Driver Among Patients Undergoing Coronary Angiography

Baseline characteristics and outcomes	No. (%)				
	All (n = 3518)	No genomic driver (n = 2009)^a^	FH variant carrier (n = 26)	High CAD PRS (n = 1191)	CHIP (n = 466)
Initial presentation					
STEMI	235 (6.7)	142 (7.1)	1 (3.8)	80 (6.7)	20 (4.3)
NSTEMI	438 (12.4)	212 (10.5)	9 (34.6)	186 (15.6)	65 (13.9)
Unstable angina	293 (8.4)	155 (7.7)	1 (3.8)	112 (9.4)	36 (7.7)
Stable CAD	1647 (46.8)	883 (44.0)	11 (42.3)	612 (51.4)	231 (49.6)
Other	905 (25.7)	617 (30.7)	4 (15.5)	201 (16.9)	114 (24.5)
CAD severity					
None	950 (27.0)	687 (34.2)	4 (15.4)	183 (15.4)	95 (20.4)
Mild	340 (9.7)	212 (10.5)	1 (3.8)	87 (7.3)	56 (12.0)
Moderate	346 (9.8)	196 (9.8)	3 (11.5)	116 (9.7)	46 (9.9)
Severe CAD	1882 (53.5)	914 (45.5)	18 (69.3)	805 (67.6)	269 (57.7)
CAD burden^b^					
Nonobstructive	686 (26.7)	408 (30.9)	4 (18.2)	203 (20.1)	102 (27.5)
1-Vessel disease	900 (35.0)	459 (34.7)	8 (36.4)	362 (35.9)	117 (31.5)
2-Vessel disease	465 (18.1)	220 (16.6)	6 (27.3)	203 (20.1)	68 (18.4)
3-Vessel disease	283 (11.0)	123 (9.3)	1 (4.5)	141 (14.0)	42 (11.3)
Left main disease	234 (9.2)	112 (8.5)	3 (13.6)	99 (9.9)	42 (11.3)
Gensini score, median (IQR)	16.0 (0-51.0)	8.0 (0-41.0)	31.0 (10.0-54.3)	32.0 (7.0-68.0)	20.0 (2.5-56.8)
Outcome					
Repeat angiogram	1286 (36.5)	633 (31.5)	15 (57.7)	539 (45.3)	182 (39.1)
Revascularization	659 (18.7)^c^	304 (15.2)^d^	9 (34.6)	296 (25.0)^e^	97 (20.8)
In-stent restenosis	286 (8.2)^f^	118 (5.9)^g^	4 (16.0)^h^	152 (12.9)^i^	37 (8.0)^j^
Heart failure	240 (15.0)^k^	128 (14.1)^l^	0	81 (15.1)^m^	54 (21.9)^n^
All-cause mortality	885 (25.3)^o^	478 (23.9)^p^	8 (30.8)	301 (25.4)^q^	172 (37.0)^r^

Abbreviations: CAD, coronary artery disease; CHIP, clonal hematopoiesis of indeterminate potential; FH, familial hypercholesterolemia; NSTEMI, non–ST elevation myocardial infarction; PRS, polygenic risk score; STEMI, ST elevation myocardial infarction.

^a^ Individuals presenting without any of the following: high CAD PRS, FH, or CHIP.

^b^ The proportion of CAD burden was calculated by excluding those who presented without angiographic CAD.

^c^ The proportion of participants who underwent revascularization out of 3506 participants.

^d^ The proportion of participants who underwent revascularization out of 2004 participants without any genomic driver.

^e^ The proportion of participants who underwent revascularization out of 1184 participants with high CAD PRS.

^f^ The proportion of participants who experienced in-stent restenosis out of 3491 participants.

^g^ The proportion of participants who experienced in-stent restenosis out of 1997 participants without any genomic driver.

^h^ The proportion of participants who experienced in-stent restenosis out of 25 participants with FH variant.

^i^ The proportion of participants who experienced in-stent restenosis out of 1179 participants with high CAD PRS.

^j^ The proportion of participants who experienced in-stent restenosis out of 463 participants with CHIP.

^k^ The proportion of participants who experienced heart failure out of 1603 participants.

^l^ The proportion of participants who experienced heart failure out of 907 participants without any genomic driver.

^m^ The proportion of participants who experienced heart failure out of 538 participants with high CAD PRS.

^n^ The proportion of participants who experienced heart failure out of 247 participants with CHIP.

^o^ The proportion of participants who experienced all-cause mortality out of 3505 participants.

^p^ The proportion of participants who experienced all-cause mortality out of 2000 participants without any genomic driver.

^q^ The proportion of participants who experienced all-cause mortality out of 1187 participants with high CAD PRS.

^r^ The proportion of participants who experienced all-cause mortality out of 465 participants with CHIP.

### Genomic Drivers of CAD Risk

Of 3518 all comers to coronary angiography, 1509 (42.9%) harbored at least 1 genomic driver of CAD. Of these, 26 patients carried FH variants: 19 (73.1%) with *LDLR* variants, 6 (23.1%) with *APOB* variants, and 1 (3.8%) with a *PCSK9* variant (eTable 7 in Supplement 1). Due to the small number of FH carriers, the estimated associations were derived from participants characterized by specific covariate profiles, predominantly male (18 of 26) and between ages 50 and 70 years (17 of 26). Additionally, we identified 1191 individuals with a high CAD PRS based on belonging to the top quintile of the distribution. In addition, 466 participants had CHIP variants in 37 driver genes, with the most common variants found in the *DNMT3A* (n = 181) and *TET2* (n = 115) genes. The median variant allele frequency was 8.7% (IQR, 5.8%-16.4%) (eTable 8 in Supplement 1).

### Genomic Drivers of Risk and CAD on Coronary Angiography

Among the 1509 participants with genomic drivers of CAD, 1246 (82.6%) had angiographic CAD: 128 (8.5%) mild, 150 (9.9%) moderate, and 968 (64.1%) severe stenosis, and 441 (35.4%) single-vessel, 405 (32.5%) multivessel, and 122 (9.8%) left main coronary disease (eTable 3 in Supplement 1). Compared with patients without a genomic driver, those with any genomic driver of CAD had increased odds of having angiographic CAD, with adjusted odds ratios (AORs) ranging from 1.61 (95% CI, 1.24-2.10; *P* = 4.09 × 10^−4^) for mild CAD to 2.94 (95% CI, 2.47-3.52; *P* = 8.57 × 10^−33^) for severe CAD. Genomic risk conferred associations with angiographic evidence of left main disease (AOR, 1.77; 95% CI, 1.31-2.40; *P* = 2.21 × 10^−4^) and an ACS presentation (AOR, 2.67; 95% CI, 2.19-3.26; *P* = 7.57 × 10^−22^) (eTable 9 in Supplement 1). We next examined whether these associations varied by each of the 3 different genomic drivers of risk.

#### Familial Hypercholesterolemia 

**Figure 1.**
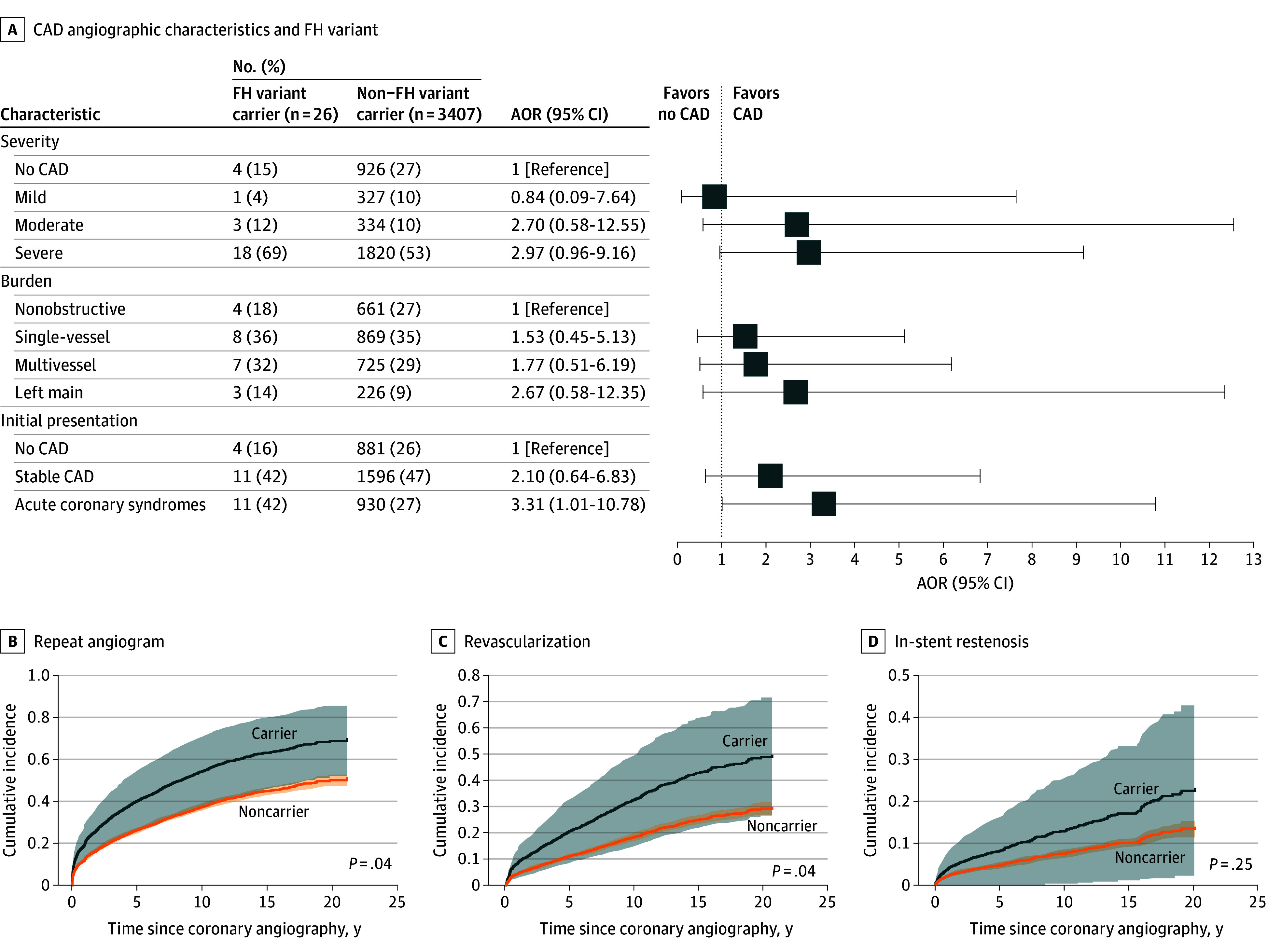
Coronary Angiography Characteristics and Risk of Outcomes by Familial Hypercholesterolemia (FH) Carrier Status A, Familial hypercholesterolemia variant status with angiographic coronary artery disease (CAD) characteristics and disease severity. The findings were calculated from a multinomial logistic regression model for categorical dependent variables and binomial logistic regression model for binary dependent variables, with age at the time of coronary angiography, sex, and genetic ancestry as defined by the first 4 genetic principal components as covariates. Multivessel was defined as a composite of 2-vessel or 3-vessel coronary lesions. Acute coronary syndromes were defined as either ST-elevation myocardial infarction, non–ST-elevation myocardial infarction, or unstable angina. Cumulative incidence curves for repeat angiogram (B), revascularization (C), and in-stent restenosis (D) by FH variant carrier status quantified using a Cox proportional hazards regression model adjusted for age at the time of coronary angiography, sex, and the first 4 principal components of genetic ancestry. The weighted numbers at risk are not shown. Shaded areas indicate 95% CIs. Revascularization was defined as a composite of percutaneous coronary intervention and coronary artery bypass graft. AOR indicates adjusted odds ratio.

Familial hypercholesterolemia variant carriers presenting for coronary angiography were more likely to have moderate to severe angiographic CAD than mild or none, with an AOR of 3.01 (95% CI, 1.18-9.28; *P* = .03). However, FH status was not associated with a higher Gensini score (β = 7.49; 95% CI, −11.66 to 26.65; *P* = .44). Individuals with FH variants were more likely to present with ACS (AOR, 3.31; 95% CI, 1.01-10.78; *P* = .05) (Figure 1A; eTable 10 in Supplement 1). These findings were also significant in a sensitivity analysis using age and sex matching (eTable 11 in Supplement 1).

#### CAD PRS

**Figure 2.**
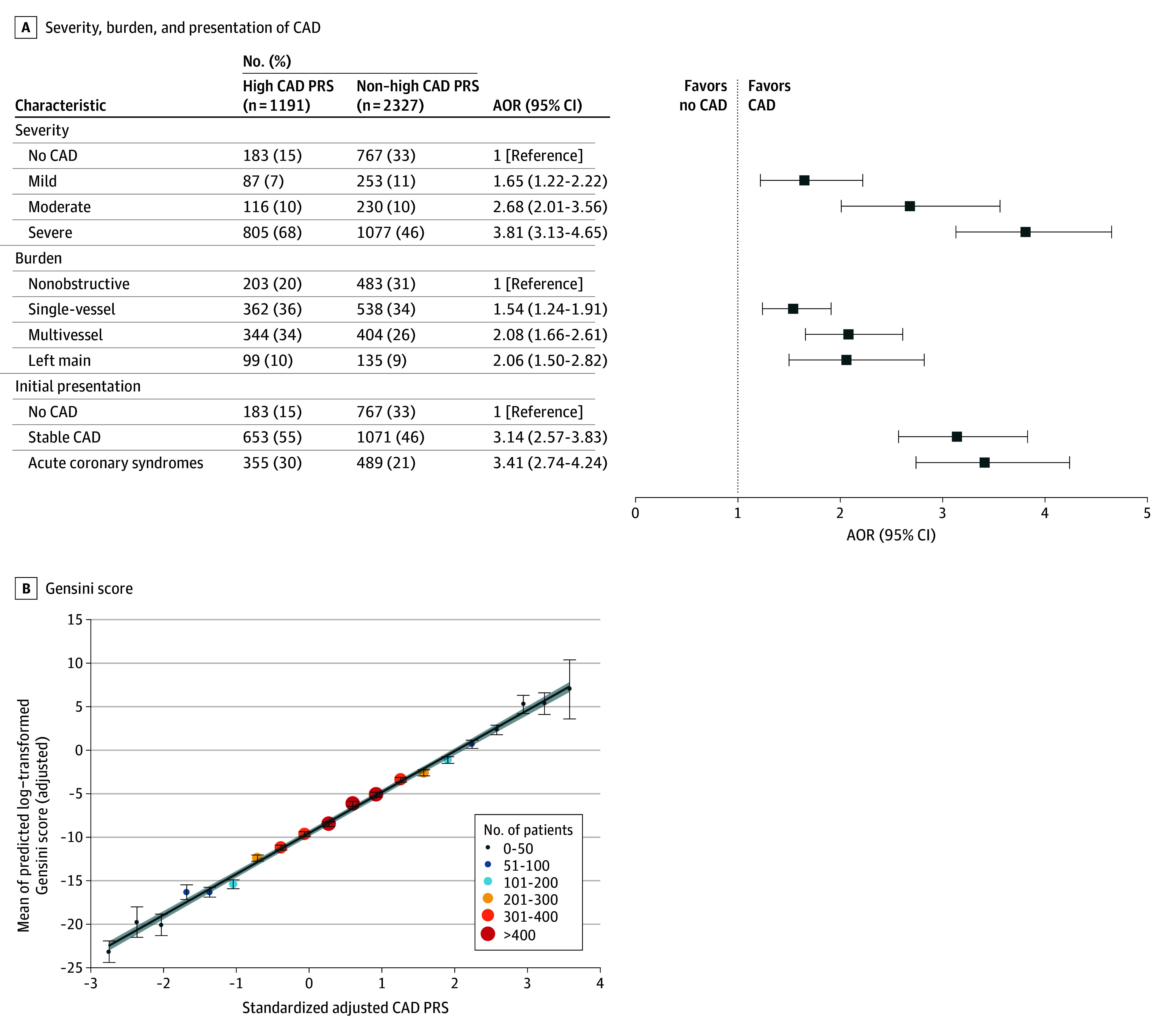
Coronary Angiography Characteristics by Coronary Artery Disease (CAD) Polygenic Risk Score (PRS) A, Association of CAD PRS with angiographic CAD by severity, burden, and presentation. The adjusted odds ratio (AOR) was calculated in a multinomial logistic regression model for categorical dependent variables and binomial logistic regression model for binary dependent variables with age at the time of coronary angiography, sex, and the first four principal components of genetic ancestry, as covariates. Multivessel was defined as a composite of 2-vessel or 3-vessel coronary lesions. Acute coronary syndromes were defined as either ST-elevation myocardial infarction, non–ST-elevation myocardial infarction, or unstable angina. Patients with initial presentation of stable CAD or acute coronary syndromes but without any angiographic evidence of CAD were reclassified into the no CAD group (reference). B, Association of CAD PRS with continuous angiographic burden of CAD as measured by the Gensini score. The mean of the estimated adjusted log-transformed Gensini score is plotted by 20 CAD PRS bins, with age at the time of coronary angiography, sex, and the first 4 principal components of genetic ancestry as covariates. Every 1-SD increase of CAD PRS equaled approximately 12.51 (95% CI, 10.94-14.07) points of the Gensini score.

A high CAD PRS was associated with the angiographic presence of CAD (AOR, 3.23; 95% CI, 2.67-3.91; *P* = 4.31×10^-25^). This did not differ significantly among patients presenting with ACS vs stable CAD (Figure 2A; eTable 12 in Supplement 1). The odds were lowest for mild CAD (AOR, 1.65; 95% CI, 1.22-2.22; *P* = 1.07 × 10^−3^) and greatest for severe CAD (AOR, 3.81; 95% CI, 3.13-4.65; *P* = 3.05 × 10^−40^). In addition to severity, a high CAD PRS was associated with the angiographic burden of CAD, with the greatest odds observed for multivessel stenosis (AOR, 2.08; 95% CI, 1.66-2.61; *P* = 1.49 × 10^−10^) followed by left main coronary stenosis (AOR, 2.06; 95% CI, 1.50-2.82; *P* = 7.85 × 10^−6^). Each SD increase in the CAD PRS was associated with a 12.51-point higher Gensini score (95% CI, 10.94-14.07; *P* < 2 × 10^−16^) (Figure 2B). Comparable changes in CAD severity, burden, and initial presentation were observed in the validation cohort (eTable 13 in Supplement 1).

#### Clonal Hematopoiesis of Indeterminate Potential 

Patients with CHIP were significantly older (mean [SD] age, 68.8 [39.8] years) when undergoing coronary angiography than those without CHIP (mean [SD] age, 61.4 [11.8] years) (*P* = 2.2 × 10^−16^). Within the limitations of statistical power, we were not able to detect associations between CHIP variant status and angiographic characteristics, even when evaluating the continuous measure of disease burden, ie, Gensini score (β = 0.82; 95% CI, −4.64 to 6.27; *P* = .77) (eTable 14 in Supplement 1). Furthermore, sensitivity analyses of only the top CHIP driver genes and large clone CHIP similarly found no conclusive associations (eTable 15 in Supplement 1).

### Genomic Drivers of CAD and Risk of Future Events After Coronary Angiography

#### Outcomes After Coronary Angiography

We investigated 5 angiographic and clinical outcomes over a mean (SD) follow-up period of 9.2 (5.8) years. A total of 1286 participants (36.5%) underwent repeat coronary angiography at 3.9 (4.2) years after their initial angiogram. Among study participants, 659 (18.7%) required revascularization at 5.3 (4.7) years and 286 (8.2%) had in-stent restenosis after 5.5 (5.2) years. There were 240 (15.0%) incident cases of heart failure observed at 6.6 (4.7) years and 885 (25.3%) cases of mortality observed at 8.4 (5.4) years.

#### FH Variants and Outcomes After Coronary Angiography

Within the FH variant carrier group (n = 26), 15 participants (57.7%) required at least 1 additional angiogram, 9 (34.6%) underwent revascularization, and 8 (30.8%) died during the follow-up period. Following initial coronary angiography, FH status was associated with an increased risk of a repeat angiogram with an adjusted hazard ratio (AHR) of 1.70 (95% CI, 1.02-2.83; *P* = .04) (Figure 1B). Similarly, FH carriers exhibited nearly twice the risk of incident revascularization (AHR, 1.97; 95% CI, 1.02-3.80; *P* = .04) at a mean period of 9.1 (6.0) years (Figure 1C). No increased risk of in-stent restenosis (AHR, 1.78; 95% CI, 0.66-4.80; *P* = .25) (Figure 1D) and mortality (AHR, 0.96; 95% CI, 0.48-1.94; *P* = .91) were observed within the limitations of statistical power. Analysis of incident heart failure was limited by the absence of outcome in FH carriers (eFigure 3 and eTable 16 in Supplement 1). A sensitivity analysis using age- and sex-matched FH carriers and controls found that associations with repeat angiography and revascularization remained numerically unchanged, while an enhanced risk for in-stent restenosis was observed (eTable 17 in Supplement 1).

#### CAD PRS and Outcomes After Coronary Angiography

**Figure 3.**
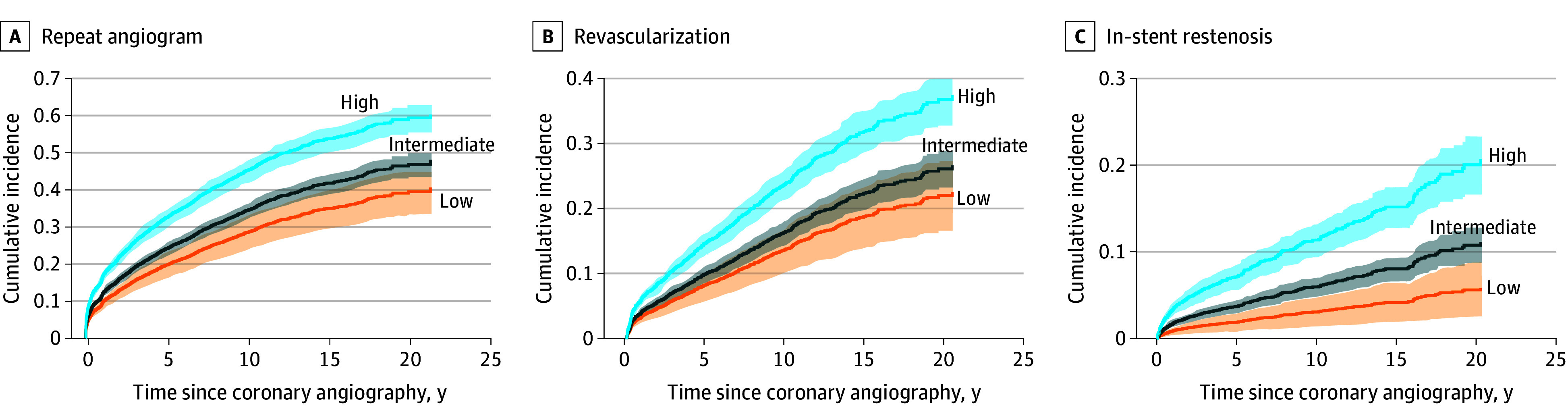
Risk of Repeat Angiogram, Revascularization, and In-Stent Restenosis by Coronary Artery Disease Polygenic Risk Score Cumulative incidence of repeat angiogram (A), revascularization (B), and in-stent restenosis (C) by coronary artery disease (CAD) polygenic risk score (PRS) group. The models were adjusted for age at the time of coronary angiography, sex, and the first 4 principal components of genetic ancestry. The weighted numbers at risk are not shown. The CAD PRS group was defined by the percentile distribution of CAD PRS, defined as low (bottom quintile), intermediate (middle three quintiles), and high (top quintile). Revascularization was defined as a composite of percutaneous coronary intervention and coronary artery bypass graft. All log-rank *P* < .001. Shaded areas indicate 95% CIs.

Patients with high CAD PRS had a 1.79-times the risk of returning for a repeat angiogram compared with those in the low-risk group (95% CI, 1.45-2.22; *P* = 5.92 × 10^−8^) (Figure 3A). The likelihood of revascularization was also significantly higher with a high CAD PRS (AHR of high vs low CAD PRS, 1.85; 95% CI, 1.37-2.50; *P* = 5.76 × 10^−5^) (Figure 3B). Additionally, patients with a high CAD PRS had a 3.89 times higher risk of in-stent restenosis (95% CI, 2.16-7.01; *P* = 6.19 × 10^−6^) (Figure 3C). Even after adjusting for baseline angiographic CAD burden (Gensini score), a high CAD PRS was still associated with the risks of repeat angiogram (AHR, 1.49; 95% CI, 1.20-1.85; *P* = 2.68 × 10^−4^), revascularization (AHR, 1.58; 95% CI, 1.17-2.15; *P* = 3.12 × 10^−3^), and in-stent restenosis (AHR, 3.26; 95% CI, 1.80-5.91; *P* = 9.72 × 10^−5^) (eFigure 4 and eTable 18 in Supplement 1). In addition, the association between the CAD PRS and heart failure (AHR, 1.34; 95% CI, 0.81-2.22; *P* = .25) or mortality (AHR, 1.18; 95% CI, 0.93-1.50; *P* = .18) was inconclusive (eFigure 5 and eTable 16 in Supplement 1). In our validation cohort of 783 participants, the high CAD PRS group (256 [32.69%]) was similarly associated with increased risks for repeat angiogram and revascularization (eTable 19 in Supplement 1).

#### CHIP Carriers and Outcomes After Coronary Angiography

**Figure 4.**
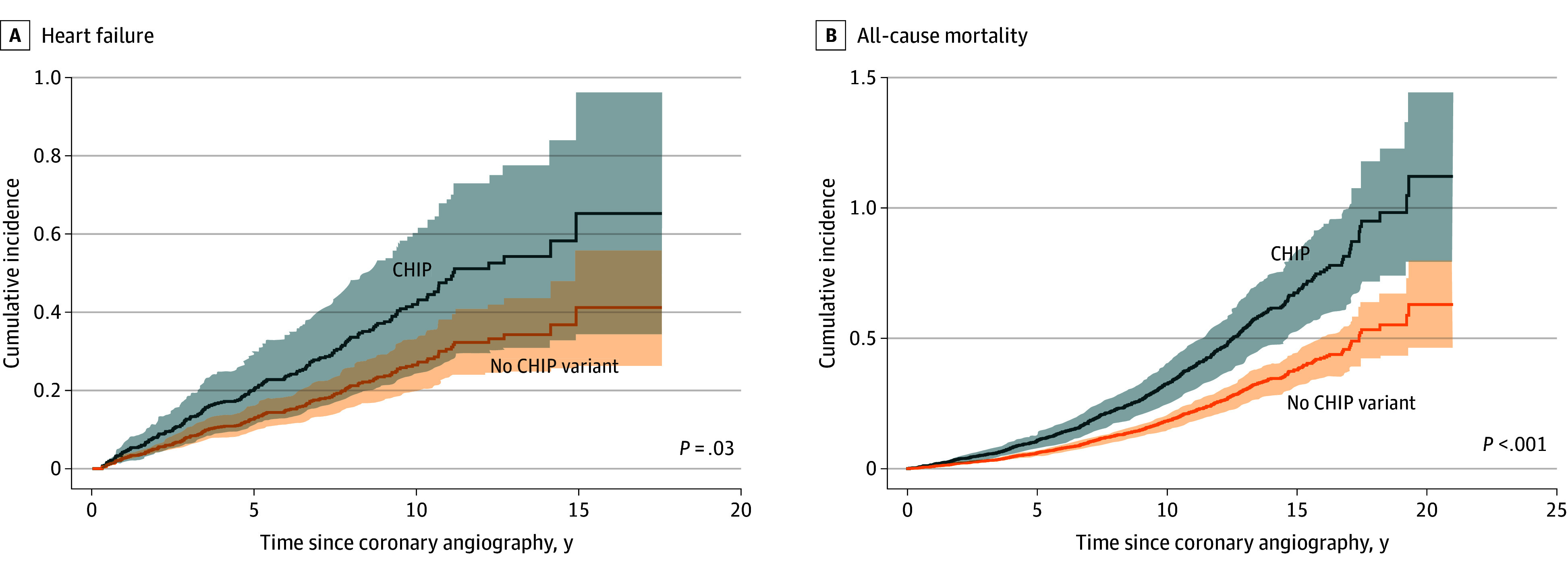
Risk of Heart Failure and All-Cause Mortality by Clonal Hematopoiesis of Indeterminate Potential (CHIP) Cumulative incidence curves of CHIP variants for heart failure (A) and all-cause mortality (B) were constructed using the time-dependent covariate Cox hazards regression model adjusted for age at the time of coronary angiography, sex, and the first 4 principal components of genetic ancestry. The weighted numbers at risk are not shown. Heart failure was defined as chronic heart failure with any ejection fraction. Shaded areas indicate 95% CIs.

While germline genomic risk (FH and CAD PRS) was associated with angiographic outcomes but not clinical outcomes (incident heart failure and mortality) after coronary angiography, the acquisition of CHIP had the opposite effect. The association between CHIP and angiographic outcomes was inconclusive: repeat angiogram (AHR, 1.07; 95% CI, 0.83-1.37; *P* = .61), revascularization (AHR, 1.18; 95% CI, 0.86-1.62; *P* = .29), and in-stent restenosis (AHR, 1.00; 95% CI, 0.60-1.70; *P* = .98) (eFigure 6 and eTable 16 in Supplement 1). However, patients with CHIP variants had increased risks of heart failure (AHR, 1.58; 95% CI, 1.04-2.40; *P* = .03) and mortality (AHR, 1.78; 95% CI, 1.47-2.16; *P* = 3.45 × 10^−9^) (Figure 4). In the sensitivity analyses, patients with non-*DNMT3A* driver genes—but not those with top CHIP driver genes or large clones—had a significantly increased risk of heart failure. In contrast, associations with mortality were consistently observed across all analyses, compared with individuals without CHIP (eTable 20 in Supplement 1).

With further adjustment for clinical risk factors of CAD, the risk trends for future outcomes persisted for a high CAD PRS and CHIP, and they were only slightly attenuated for FH (eTable 16 in Supplement 1). Additionally, although most participants (69.86%) were sequenced after their first angiogram (eFigure 7 in Supplement 1) and thus could not experience any fatal outcome during that period, the association between genomic drivers and mortality risk remained unchanged when excluding patients whose blood sequencing occurred more than 1 year after their coronary angiography (eTable 21 in Supplement 1).

## Discussion

In this study of 3518 patients presenting for coronary angiography, 43% had at least 1 genomic risk factor (FH, high CAD PRS, or CHIP variant). An association was observed for CAD PRS and FH, but not CHIP, with angiographic CAD severity and burden. The presence of FH or a high CAD PRS was also associated with future angiographic outcomes, including repeat angiogram, revascularization, and in-stent restenosis, even after adjusting for baseline disease burden. CHIP variants were independently associated with incident heart failure and death, but not with angiographic outcomes. To our knowledge, this is the first study evaluating the association of germline and somatic genomic drivers of CAD with disease presentation on coronary angiography and their role in estimating future outcomes even after disease is identified on coronary angiography.

Most CAD genomic studies focus on binary clinical diagnoses. Phenotyping coronary atherosclerosis using coronary angiography provides a mechanistic understanding of how genomic risk affects disease. Herein, we observed that monogenic and polygenic risks are associated with atherosclerotic severity and burden, using multiple clinical measures (maximal severity of stenosis, number of diseased vessels, and burden). These findings corroborate previous studies linking FH with the degree and number of coronary stenoses noted on angiograms.^34,35^ Our study also demonstrates a similar association of the CAD PRS with the number of obstructive vessels, consistent with a previously reported finding.^36^ The CAD PRS association with plaque burden could be modeled using a continuous measure—each SD of the CAD PRS is associated with a 12.5-point higher Gensini score. This finding is consistent with prior studies showing a positive association of the CAD PRS with plaque volume on coronary computed tomographic angiography.^37,38^ In addition to an increase in plaque burden and severity, germline genomic drivers were more strongly associated with acute disease presentations than with no CAD or stable CAD, as demonstrated by the higher odds for ACS. These findings are consistent with a previously reported association of CAD PRS with high-risk plaque features on coronary computed tomography angiography, which are predictors of plaque rupture.^37^ Familial hypercholesterolemia variants operate via increasing low-density lipoprotein cholesterol levels, resulting in increased plaque burden and severity and predisposing to rupture due to lipid-rich plaque.^39^ The CAD PRS operates via multiple pathways, including low-density lipoprotein cholesterol, inflammation, and others that remain unknown.^40^ This study, together with prior ones using coronary computed tomographic angiography,^37,38^ provides robust evidence that polygenic risk primarily manifests in an increased plaque volume. Unlike germline genomic risk, we could not detect statistically significant associations of somatic CHIP-driver variants with CAD angiographic characteristics within the limitations of statistical power. At least 1 prior study has found an association of CHIP with left main coronary stenosis, which we did not observe in this study.^41^ Given the limitations of coronary angiography in assessing the biological properties of plaques, future studies using intravascular imaging, such as optical coherence tomography, could better characterize plaque biologic characteristics among individuals with genomic risk.^42,43^

This study also underscores the added prognostic value of genomic risk assessments at the time of coronary angiography in forecasting future outcomes. While genomics has conventionally been perceived as most helpful in primary CAD prevention, recent evidence shows a strong ability to even predict risk of recurrent events after a CAD diagnosis.^17,44,45^ We observed that the germline genomic risk at the time of coronary angiography may be predictive of repeat angiogram, revascularization, and in-stent restenosis, even after adjusting for disease burden. The mechanistic biologic factors behind how germline risk augments the risk of in-stent restenosis remains an open-ended question and warrants further investigation. Nonetheless, this suggests that genomic risk might drive an accelerated progression of atherosclerosis even in the context of treatment. As such, genomic risk information might be helpful in guiding secondary prevention strategies, even after CAD is diagnosed on coronary angiography, such as with more aggressive treatment goals for individuals at the highest risk of progression due to the underlying genomic profile. One prior study reported that CAD PRS is associated with all-cause mortality after coronary angiography among patients with no CAD.^46^ In this study with a larger sample size and longer follow-up, we did not observe any association of CAD PRS with heart failure or mortality after coronary angiography. In addition, patients with CHIP variants did not have an increased risk of angiographic outcomes, but did have an increased risk of heart failure and mortality. This might suggest that CHIP does not strongly estimate accelerated atherosclerosis progression to an extent that it would be clinically meaningful to be observed with the revascularization risk of in-stent restenosis, although this would need to be validated in larger studies. The association of CHIP with heart failure and mortality has been established from multiple prior studies,^22,47,48,49^ and it was observed also in this study following coronary angiography. In this study, we assessed each genomic driver individually; however, ongoing work aims to assess whether these genomic drivers act cooperatively to influence adverse outcomes, considering the polygenic liability of monogenic and somatic variants.^14,19^

### Limitations

Our findings must be interpreted within the context of potential limitations. First, the estimated associations for FH carriers were based on a small sample size and may require extrapolation when applied to broader populations with different combinations of covariates. Second, while coronary angiography provides valuable insights into CAD, its ability to fully assess plaque presence, biologic factors, and extent is limited. Future research using intravascular imaging could better evaluate the influence of genomic risk on plaque morphologic characteristics. Third, the adjudication of angiographic phenotypes, despite expert review, remains subject to potential misclassification and variability in interrater reliability.^50,51,52,53^ Fourth, CHIP ascertainment was within a 10-year window prior to coronary angiography. As such, individuals who developed CHIP after biobank enrollment but before angiography in our study could be missed. Fifth, as analyses were restricted to individuals undergoing coronary angiography, the potential for selection or collider bias could not be fully ruled out and results should be interpreted with caution only within the context of patients presenting for coronary angiography. Sixth, the study population consisted primarily of individuals of White race. Additional research is needed to establish generalizability of the findings in diverse ancestries, especially as the CAD PRS notably underperforms in individuals of African ancestry.^11^

## Conclusions

This cohort study observed that monogenic and polygenic risk of CAD were associated with an ACS presentation, with severity and burden of atherosclerotic plaque on coronary angiography, and with a future risk of repeat angiography, revascularization, and in-stent restenosis. CHIP variant status was associated with incident heart failure and mortality among patients undergoing coronary angiography.

## Notes

Supplement 1. **eMethods.** Detailed Methods **eFigure 1.** Study Flowchart **eFigure 2.** Clonal Hematopoiesis of Indeterminate Potential (CHIP) Curation Flowchart **eFigure 3.** Univariable Cumulative Incidence Curves of Outcomes by Familial Hypercholesterolemia (FH) Variant Status **eFigure 4.** Cumulative Incidence of Outcomes by CAD PRS Adjusted for Age, Sex, Genetic Ancestry, and Angiographic Burden of CAD **eFigure 5.** Univariable Cumulative Incidence Curves of Outcomes by Coronary Artery Disease (CAD) Polygenic Risk Score (PRS) **eFigure 6.** Univariable Cumulative Incidence Curves of Outcomes by Clonal Hematopoiesis of Indeterminate Potential (CHIP) **eFigure 7.** Differences in Age at Sample Collection for Genotyping Vs Age at First Coronary Angiography **eTable 1.** Baseline Characteristics of Study Participants **eTable 2.** Comparison of Demographic Characteristics between Coronary Angiography Cohort and the Remaining MGBB Participants **eTable 3.** Angiographic Characteristics and Outcomes Based on the Presence of Any Genomic Drivers **eTable 4.** Angiographic Characteristics and Outcomes by Familial Hypercholesterolemia (FH) Variant Status **eTable 5.** Angiographic Characteristics and Outcomes by Coronary Artery Disease (CAD) Polygenic Risk Score (PRS) Group **eTable 6.** Angiographic Characteristics and Outcomes by Clonal Hematopoiesis of Indeterminate Potential (CHIP) **eTable 7.** List of Pathogenic or Likely Pathogenic Variants of Familial Hypercholesterolemia in the Study Cohort (N = 26) **eTable 8.** Frequency of Clonal Hematopoiesis of Indeterminate Potential Driver Genes in the Study Cohort (N = 466) **eTable 9.** Univariable and Multivariable-Adjusted Associations of Coronary Angiography Characteristics With the Presence of Genomic Drivers **eTable 10.** Univariable and Multivariable-Adjusted Associations of Coronary Angiography Characteristics With Familial Hypercholesterolemia (FH) Variant Status **eTable 11.** Age and Sex-Matched Univariable and Multivariable-Adjusted Associations of Coronary Angiography Characteristics With Familial Hypercholesterolemia (FH) Variant Status **eTable 12.** Univariable and Multivariable-Adjusted Associations of Coronary Angiography Characteristics With Coronary Artery Disease (CAD) Polygenic Risk Score (PRS) **eTable 13.** Univariable and Multivariable-Adjusted Associations of Coronary Angiography Characteristics With Coronary Artery Disease (CAD) Polygenic Risk Score (PRS) in the Replication Cohort (N = 783) **eTable 14.** Univariable and Multivariable-Adjusted Associations of Coronary Angiography Characteristics With Clonal Hematopoiesis of Indeterminate Potential (CHIP) **eTable 15.** Univariable and Multivariable-Adjusted Associations of Coronary Angiography Characteristics With Clonal Hematopoiesis of Indeterminate Potential (CHIP) by Driver Genes and Clone Size **eTable 16.** Univariable and Multivariable-Adjusted Cox Regression Models of Familial Hypercholesterolemia (FH) Variant, Coronary Artery Disease (CAD) Polygenic Risk Score (PRS), and Clonal Hematopoiesis of Indeterminate Potential (CHIP) **eTable 17.** Age and Sex-Matched Univariable and Multivariable-Adjusted Cox Regression Models of Familial Hypercholesterolemia (FH) Variant **eTable 18.** Multivariable-Adjusted Cox Regression Models of Coronary Artery Disease (CAD) Polygenic Risk Score (PRS) Accounting for Baseline Disease Burden **eTable 19.** Univariable and Multivariable-Adjusted Cox Regression Models of Coronary Artery Disease (CAD) Polygenic Risk Score (PRS) in the Replication Cohort (N = 783) **eTable 20.** Cox Regression Models for All-Cause Mortality by Clonal Hematopoiesis of Indeterminate Potential (CHIP) by Driver Genes and Clone Size **eTable 21.** Cox Regression Models for All-Cause Mortality by CAD PRS and Clonal Hematopoiesis of Indeterminate Potential Restricted on Individuals Sequenced Either Before or Within a Maximum of 1 Year Before Their First Coronary Angiography
eReferences

Supplement 2.
Data Sharing Statement
