# Supplementary material for: Genomic Drivers of Coronary Artery Disease and Risk of Future Outcomes After Coronary Angiography
Source: JAMA Netw Open. 2025 Jan 21;8(1):e2455368. doi: 10.1001/jamanetworkopen.2024.55368 (PMC11751748; doi:10.1001/jamanetworkopen.2024.55368)
Supplement: Supplement 1. — eMethods. Detailed Methods eFigure 1. Study Flowchart eFigure 2. Clonal Hematopoiesis of Indeterminate Potential (CHIP) Curation Flowchart eFigure 3. Univariable Cumulative Incidence Curves of Outcomes by Familial Hypercholesterolemia (FH) Variant Status eFigure 4. Cumulative Incidence of Outcomes by CAD PRS Adjusted for Age, Sex, Genetic Ancestry, and Angiographic Burden of CAD eFigure 5. Univariable Cumulative Incidence Curves of Outcomes by Coronary Artery Disease (CAD) Polygenic Risk Score (PRS) eFigure 6. Univariable Cumulative Incidence Curves of Outcomes by Clonal Hematopoiesis of Indeterminate Potential (CHIP) eFigure 7. Differences in Age at Sample Collection for Genotyping Vs Age at First Coronary Angiography eTable 1. Baseline Characteristics of Study Participants eTable 2. Comparison of Demographic Characteristics between Coronary Angiography Cohort and the Remaining MGBB Participants eTable 3. Angiographic Characteristics and Outcomes Based on the Presence of Any Genomic Drivers eTable 4. Angiographic Characteristics and Outcomes by Familial Hypercholesterolemia (FH) Variant Status eTable 5. Angiographic Characteristics and Outcomes by Coronary Artery Disease (CAD) Polygenic Risk Score (PRS) Group eTable 6. Angiographic Characteristics and Outcomes by Clonal Hematopoiesis of Indeterminate Potential (CHIP) eTable 7. List of Pathogenic or Likely Pathogenic Variants of Familial Hypercholesterolemia in the Study Cohort (N = 26) eTable 8. Frequency of Clonal Hematopoiesis of Indeterminate Potential Driver Genes in the Study Cohort (N = 466) eTable 9. Univariable and Multivariable-Adjusted Associations of Coronary Angiography Characteristics With the Presence of Genomic Drivers eTable 10. Univariable and Multivariable-Adjusted Associations of Coronary Angiography Characteristics With Familial Hypercholesterolemia (FH) Variant Status eTable 11. Age and Sex-Matched Univariable and Multivariable-Adjusted Associations of Coronary Angiography Characteristics With [file jamanetwopen-e2455368-s001.pdf]

Supplementary Online Content

Supriami K, Urbut SM, Tello-Ayala JR, et al. Genomic drivers of coronary artery disease and risk of future outcomes after coronary angiography. *JAMA Netw Open*. 2025;8(1):e2455368. doi:10.1001/jamanetworkopen.2024.55368

eMethods. Detailed Methods
eFigure 1. Study Flowchart
eFigure 2. Clonal Hematopoiesis of Indeterminate Potential (CHIP) Curation Flowchart
eFigure 3. Univariable Cumulative Incidence Curves of Outcomes by Familial Hypercholesterolemia (FH) Variant Status
eFigure 4. Cumulative Incidence of Outcomes by CAD PRS Adjusted for Age, Sex, Genetic Ancestry, and Angiographic Burden of CAD
eFigure 5. Univariable Cumulative Incidence Curves of Outcomes by Coronary Artery Disease (CAD) Polygenic Risk Score (PRS)
eFigure 6. Univariable Cumulative Incidence Curves of Outcomes by Clonal Hematopoiesis of Indeterminate Potential (CHIP) Mutation
eFigure 7. Differences in Age at Sample Collection for Genotyping Versus Age at First Coronary Angiography
eTable 1. Baseline Characteristics of Study Participants
eTable 2. Comparison of Demographic Characteristics between Coronary Angiography Cohort and the Remaining MGBB Participants
eTable 3. Angiographic Characteristics and Outcomes Based on the Presence of Any Genomic Drivers
eTable 4. Angiographic Characteristics and Outcomes by Familial Hypercholesterolemia (FH) Variant Status
eTable 5. Angiographic Characteristics and Outcomes by Coronary Artery Disease (CAD) Polygenic Risk Score (PRS) Group
eTable 6. Angiographic Characteristics and Outcomes by Clonal Hematopoiesis of Indeterminate Potential (CHIP) Mutation
eTable 7. List of Pathogenic or Likely Pathogenic Variants of Familial Hypercholesterolemia in the Study Cohort (N = 26)
eTable 8. Frequency of Clonal Hematopoiesis of Indeterminate Potential Driver Genes in the Study Cohort (N = 466)
eTable 9. Univariable and Multivariable-Adjusted Associations of Coronary Angiography Characteristics With the Presence of Genomic Drivers
eTable 10. Univariable and Multivariable-Adjusted Associations of Coronary Angiography Characteristics With Familial Hypercholesterolemia (FH) Variant Status
eTable 11. Age and Sex-Matched Univariable and Multivariable-Adjusted Associations of Coronary Angiography Characteristics With Familial Hypercholesterolemia (FH) Variant Status
eTable 12. Univariable and Multivariable-Adjusted Associations of Coronary Angiography Characteristics With Coronary Artery Disease (CAD) Polygenic Risk Score (PRS)
eTable 13. Univariable and Multivariable-Adjusted Associations of Coronary Angiography Characteristics With Coronary Artery Disease (CAD) Polygenic Risk Score (PRS) in the Replication Cohort (N=783)
eTable 14. Univariable and Multivariable-Adjusted Associations of Coronary Angiography Characteristics With Clonal Hematopoiesis of Indeterminate Potential (CHIP) Mutation
eTable 15. Univariable and Multivariable-Adjusted Associations of Coronary Angiography Characteristics With Clonal Hematopoiesis of Indeterminate Potential (CHIP) Mutation by Driver Genes and Clone Size
eTable 16. Univariable and Multivariable-Adjusted Cox Regression Models of Familial Hypercholesterolemia (FH) Variant, Coronary Artery Disease (CAD) Polygenic Risk Score (PRS), and Clonal Hematopoiesis of Indeterminate Potential (CHIP) Mutation
eTable 17. Age and Sex-Matched Univariable and Multivariable-Adjusted Cox Regression Models of Familial Hypercholesterolemia (FH) Variant
eTable 18. Multivariable-Adjusted Cox Regression Models of Coronary Artery Disease (CAD) Polygenic Risk Score (PRS) Accounting for Baseline Disease Burden
eTable 19. Univariable and Multivariable-Adjusted Cox Regression Models of Coronary Artery Disease (CAD) Polygenic Risk Score (PRS) in the Replication Cohort (N=783)
eTable 20. Cox Regression Models for All-Cause Mortality by Clonal Hematopoiesis of Indeterminate Potential (CHIP) by Driver Genes and Clone Size
eTable 21. Cox Regression Models for All-Cause Mortality by CAD PRS and Clonal Hematopoiesis of Indeterminate Potential Restricted on Individuals Sequenced Either Before or Within a Maximum of 1 Year Before Their First Coronary Angiography
eReferences

This supplementary material has been provided by the authors to give readers additional information about their work.

© 2025 Supriami K et al. *JAMA Network Open.*

eMethods. Detailed Methods

Computation, Adjustment, and Standardization of CAD PRS

Within the MGB Biobank population with available genetic data (n=53,125), the unadjusted raw coronary artery disease (CAD) polygenic risk score (PRS_{raw}) was computed from GPS_{mult} (PGS003725) weights file, and then then adjusted for population stratification by using the top 20 principal components (PCs) of genetic ancestry. We used a linear regression model to regress these PCs with the unadjusted raw polygenic score (PRS_{raw} ~ PC1 + PC2 + PC3 + … + PC20) and then subtracted the predicted score (PRS_{pred}) from this model fit from the raw polygenic score to get an adjusted PRS (PRS_{raw} - PRS_{pred}). Next, we scaled or standardized these residuals so that the mean is 0 with a standard deviation of 1 to center the population-specific polygenic distributions and enable us to perform better comparison of genetic risk within the MGB Biobank. We ranked the scaled adjusted CAD PRS in the MGB Biobank into 100 groups (percentiles) from lowest to highest PRS value. We classified participants as having high ($81\text{-}100^{th}$ percentile), intermediate ($21\text{-}80^{th}$ percentile), and low ($1\text{-}20^{th}$ percentile) CAD PRS, consistent with prior studies.[1] Finally, we identified how many individuals from the current study cohort of 3,158 patients fell into the high CAD PRS group in the overall MGB Biobank population.[2,3]

Ascertainment of Demographic Information and Traditional Cardiovascular Risk Factors

Race was self-reported as White, Black, Asian, Other, or recorded as not available (NA) if not reported. Clinical risk factors for cardiovascular disease were collected from pooled electronic health records obtained from the Research Patient Data Registry (RPDR), which represented the study participants' latest clinical comorbidities before they had their first coronary angiography at Massachusetts General Hospital. Hypertension was defined as either systolic blood pressure (SBP) ≥130 mmHg, diastolic blood pressure (DBP) ≥80 mmHg, physician-adjudicated diagnosis of hypertension, a diagnosis retrieved from diagnostic codes, inpatient records, and All Patient Refined Diagnostic-Related Groups classifications, or a prescription of anti-hypertension medications. Hypercholesterolemia status was curated based on the presence of low-density lipoprotein-cholesterol (LDL-C) ≥130 mg/dL or any prescription of lipid-lowering agents. We defined diabetes mellitus type 2 as either Hemoglobin A1c (HbA1c) ≥6.5%, a diagnosis of diabetes mellitus type 2 from physicians, or any diagnosis containing the term diabetes mellitus ascertained from physician notes, diagnostic codes, inpatient records, and All Patient Refined Diagnostic-Related Groups classifications. Current smoker was defined as ever answering yes to smoking tobacco without any documented records of cessation, as described previously.[4,5]

© 2025 Supriami K et al. *JAMA Network Open.*

eFigure 1. Study Flowchart

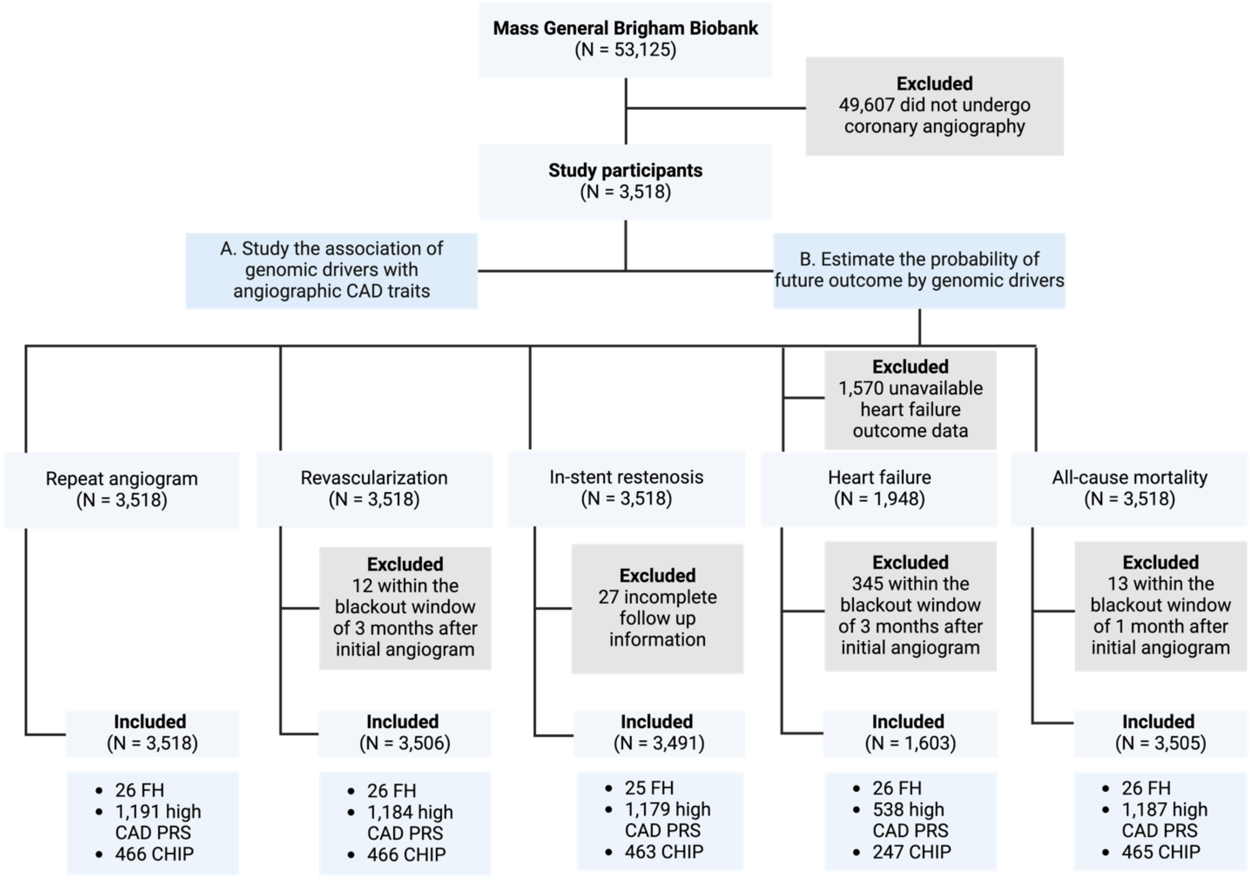

© 2025 Supriami K et al. *JAMA Network Open.*

eFigure 2. Clonal Hematopoiesis of Indeterminate Potential (CHIP) Curation Flowchart

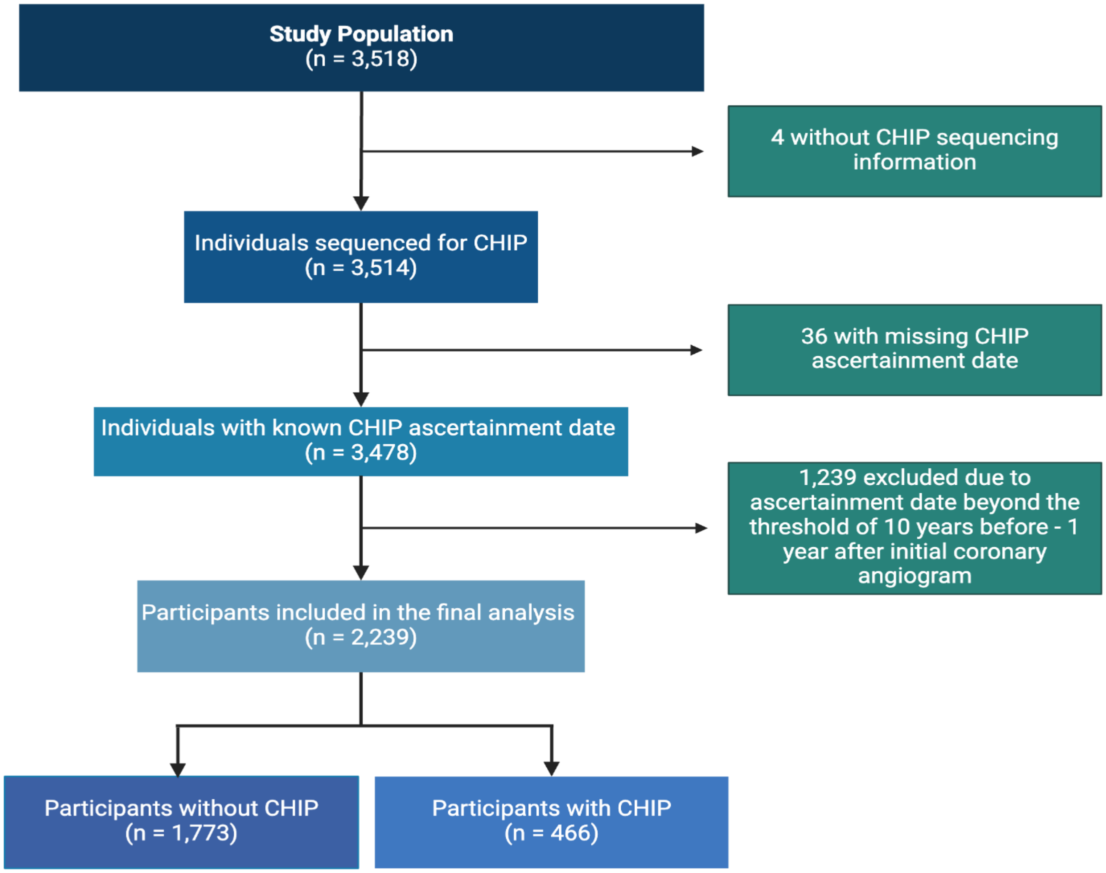

© 2025 Supriami K et al. *JAMA Network Open.*

eFigure 3. Univariable Cumulative Incidence Curves of Outcomes by Familial Hypercholesterolemia (FH) Variant Status

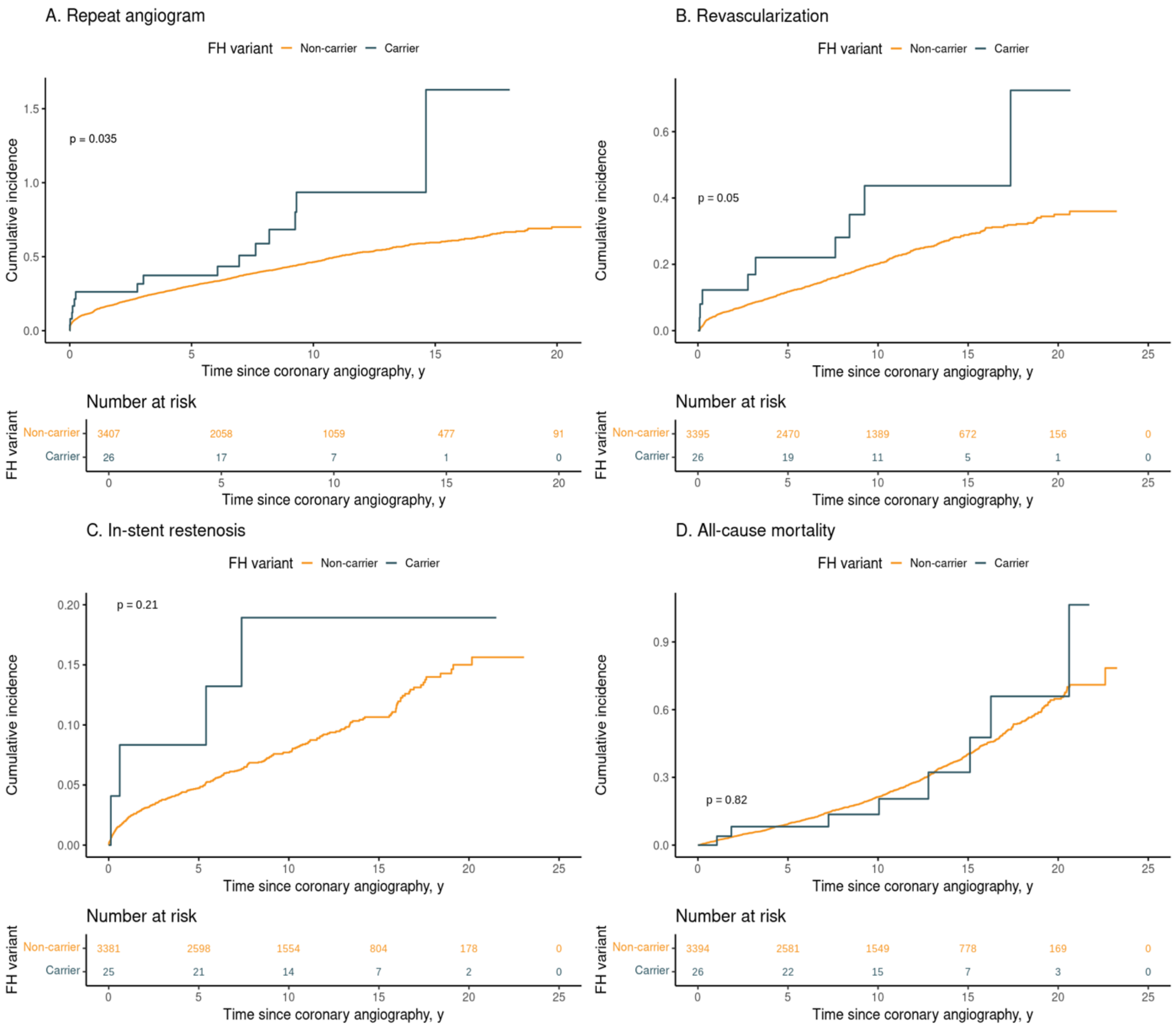

Cumulative incidence curves for repeat angiogram (A), revascularization (B), in-stent restenosis (C), and all-cause mortality (D) were constructed using the Kaplan-Meier method. *P-value* was calculated from the unadjusted log-rank test. Revascularization was a composite outcome of percutaneous coronary intervention and coronary artery bypass graft. The cumulative incidence curve for heart failure (HF) by FH variant could not be generated due to absence of incident HF in this group.

© 2025 Supriami K et al. *JAMA Network Open.*

eFigure 4. Cumulative Incidence of Outcomes by CAD PRS Adjusted for Age, Sex, Genetic Ancestry, and Angiographic Burden of CAD

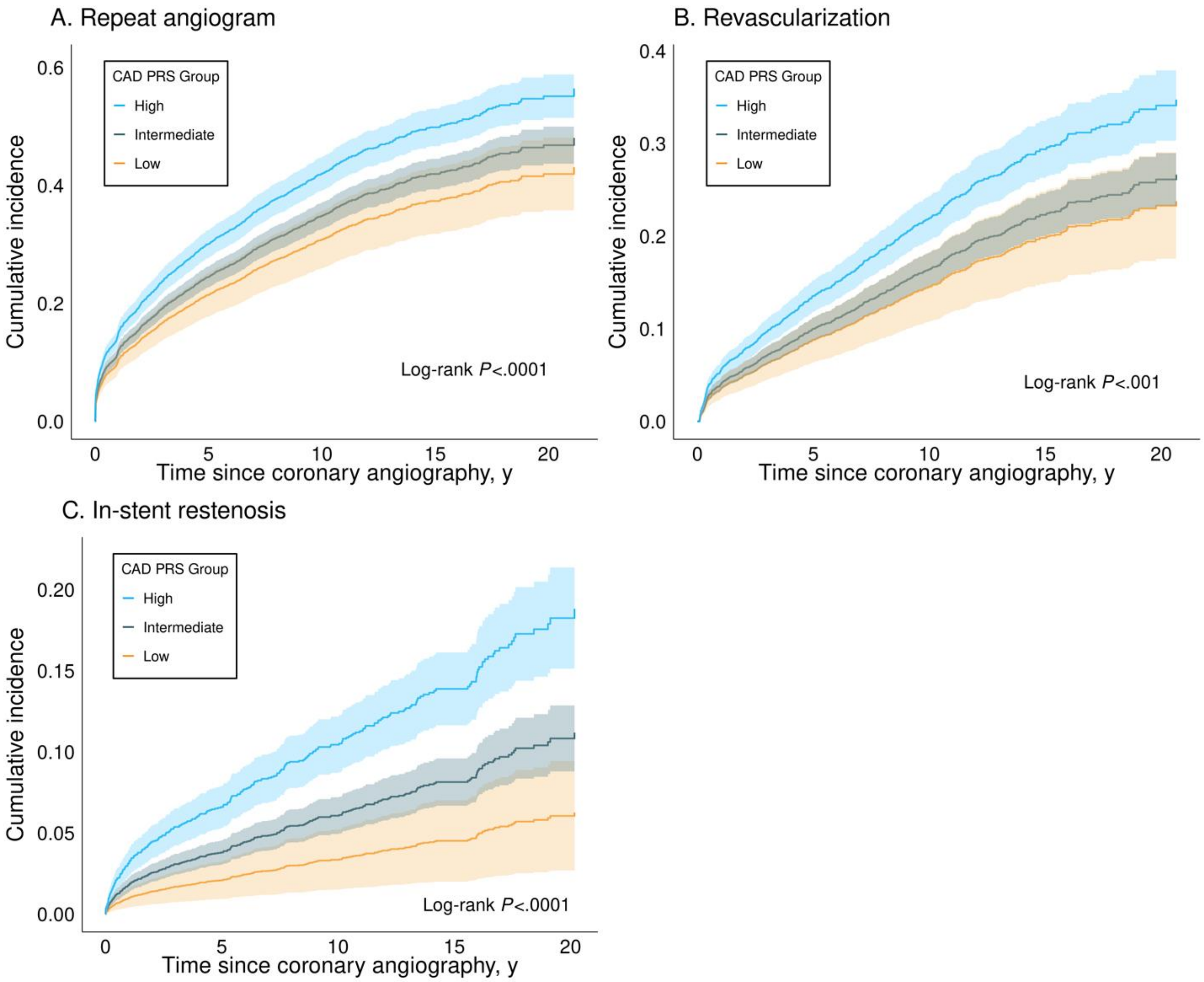

Cumulative incidence of repeat angiogram (A), revascularization (B), and in-stent restenosis (C) by coronary artery disease (CAD) polygenic risk score (PRS) group. The models were adjusted for age at the time of coronary angiography, sex, genetic ancestry as measured by the first 4 principal components of genetic ancestry, and continuous angiographic burden of CAD as quantified by the Gensini score. The CAD PRS group was defined by the percentile distribution of CAD PRS, defined as low (bottom quintile), intermediate (middle three quintiles), and high (top quintile). Revascularization was defined as a composite of percutaneous coronary intervention and coronary artery bypass graft.

© 2025 Supriami K et al. *JAMA Network Open.*

eFigure 5. Univariable Cumulative Incidence Curves of Outcomes by Coronary Artery Disease (CAD) Polygenic Risk Score (PRS)

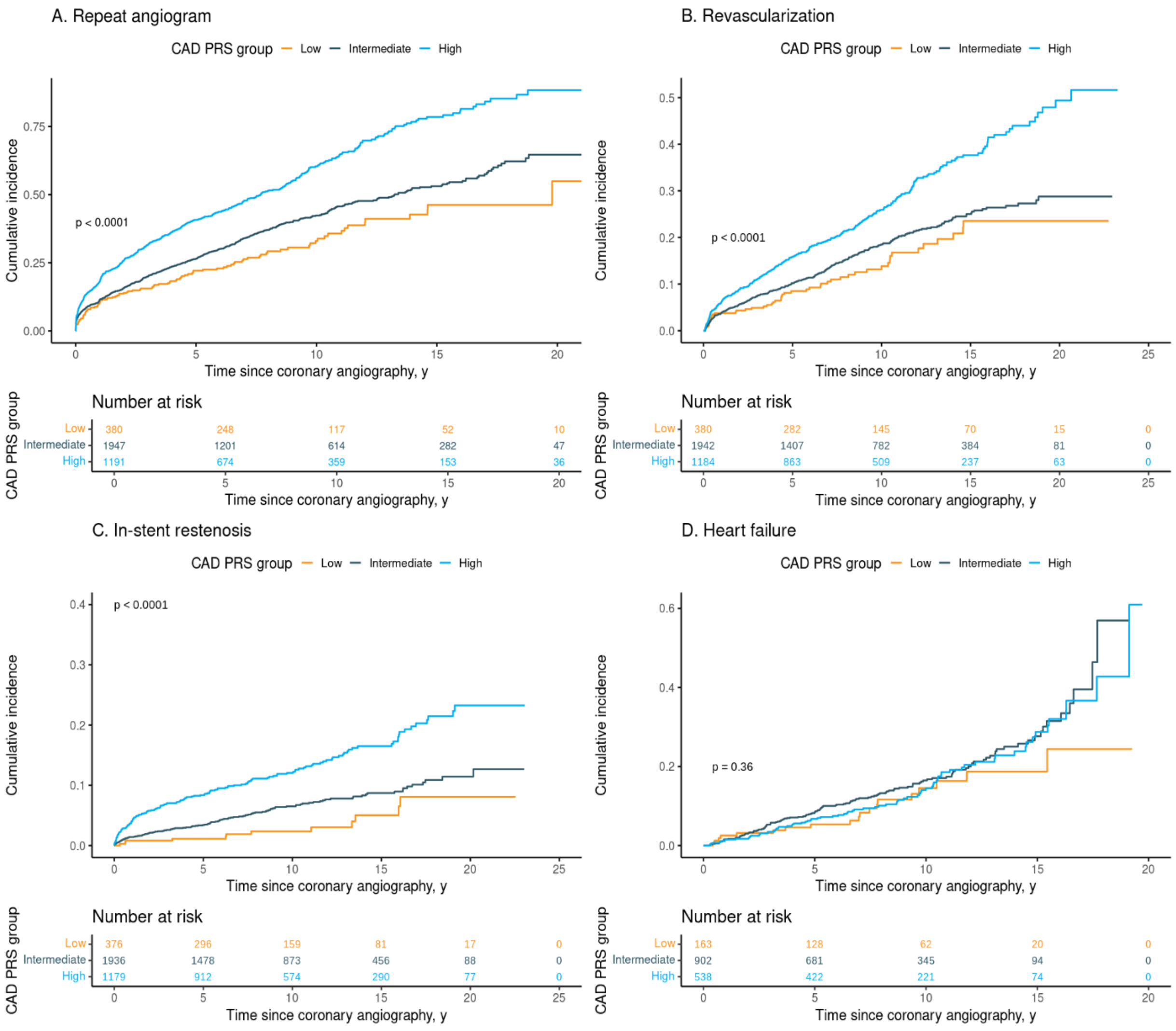

© 2025 Supriami K et al. *JAMA Network Open.*

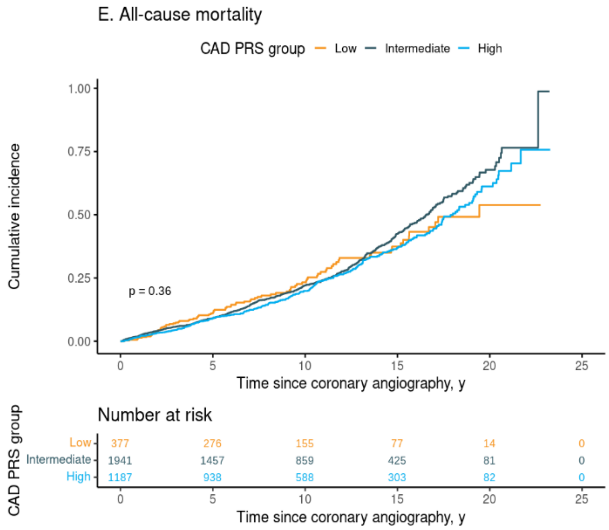

Cumulative incidence curves for repeat angiogram (A), revascularization (B), in-stent restenosis (C), heart failure (D), and all-cause mortality (E) were constructed using the Kaplan-Meier method. *P-value* was calculated from the unadjusted log-rank test. The CAD PRS group was defined by the percentile distribution of CAD PRS, defined as low (bottom quintile), intermediate (middle three quintiles), and high (top quintile). Revascularization was a composite outcome of percutaneous coronary intervention and coronary artery bypass graft. Heart failure was defined as chronic heart failure with any ejection fraction.

eFigure 6. Univariable Cumulative Incidence Curves of Outcomes by Clonal Hematopoiesis of Indeterminate Potential (CHIP) Mutation

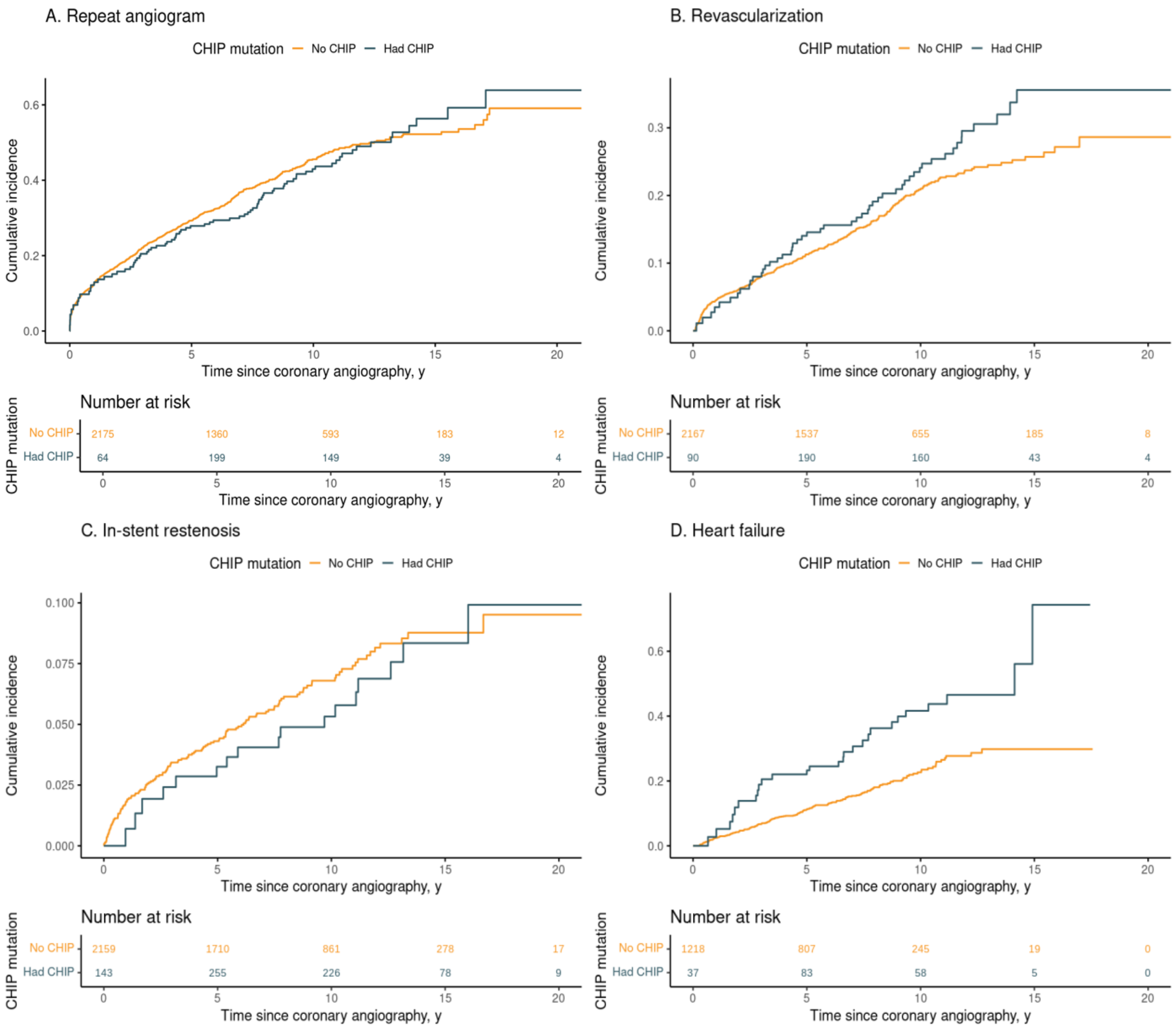

© 2025 Supriami K et al. *JAMA Network Open.*

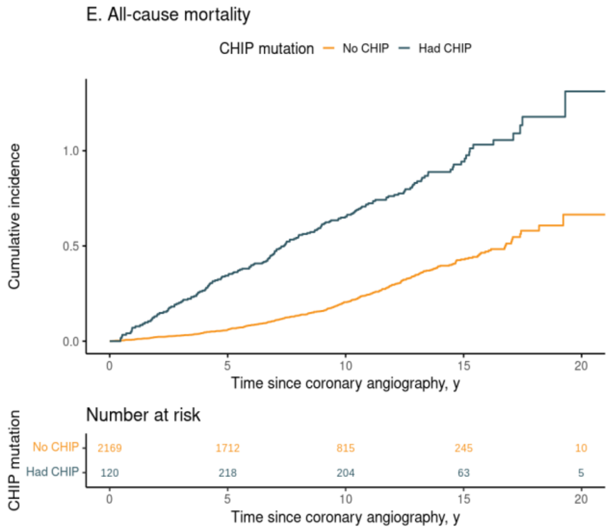

Cumulative incidence curves for repeat angiogram (A), revascularization (B), in-stent restenosis (C), heart failure (D), and all-cause mortality (E) were constructed using the Kaplan-Meier method with adjustment of CHIP as a time-dependent variable. Revascularization was a composite outcome of percutaneous coronary intervention and coronary artery bypass graft. Heart failure was defined as chronic heart failure with any ejection fraction. The number at risk table for CHIP should be interpreted with caution since the participants did not have the 'CHIP' risk factor until they had the laboratory examination. Hence, the risk factor status of these patients changed over time.

© 2025 Supriami K et al. *JAMA Network Open.*

eFigure 7. Differences in Age at Sample Collection for Genotyping versus Age at First Coronary Angiography

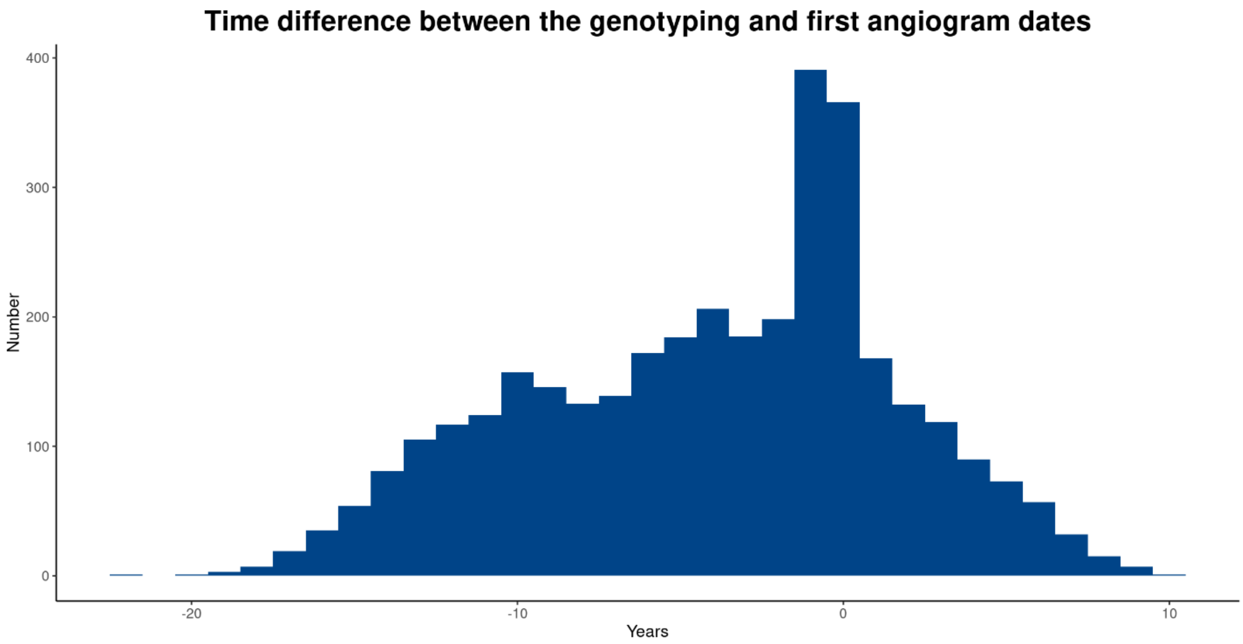

Out of 3,518 study participants, 2,458 (69.9%) were enrolled to the biobank and genotyped after their first coronary angiography date. There were 365 participants who were genotyped on the same day as their first coronary angiography.

© 2025 Supriami K et al. *JAMA Network Open.*

eTable 1. Baseline Characteristics of Study Participants

Baseline Characteristics	Patients (n = 3,518)
**Female, n (%)**	1,051 (29.9)
**Age at angiogram, median (IQR)**	64.0 (55.0-72.0)
**Height, median (IQR)**	173.0 (165.0-180.0)
**Weight, median (IQR)**	84.8 (73.5-98.9)
**BMI, median (IQR)**	28.4 (25.3-32.5)
**Race**	
White, n (%)	3,152 (89.6)
Black, n (%)	135 (3.8)
Asian, n (%)	38 (1.1)
Other, n (%)	120 (3.4)
NA, n (%)	73 (2.1)
**Comorbidities**	
Hypertension, n (%)	3,181 (90.4)
Hypercholesterolemia, n (%)	2,686 (76.4)
Diabetes mellitus type 2, n (%)	1,478 (42.0)
Current smoking, n (%)	84 (2.4)
**Genomic driver**	
FH variant, n (%)[a]	26 (0.8)
CAD PRS, mean (SD)	1.7 (6.1)
High CAD PRS, n (%)	1,191 (33.8)
CHIP variant, n (%)[b]	466 (20.8)

Abbreviations: BMI, body mass index; CAD, coronary artery disease; CHIP, clonal hematopoiesis of indeterminate potential; FH, familial hypercholesterolemia; NA, not available; PRS, polygenic risk score.
[a] The proportion of people with FH variant out of individuals with available whole exome sequencing data used for FH analysis (n=3,433).
[b] The proportion of people with CHIP after filtering the CHIP ascertainment date within the range of 10 years before and 1 year after the initial angiogram (n=2,239).

© 2025 Supriami K et al. *JAMA Network Open.*

eTable 2. Comparison of Demographic Characteristics between Coronary Angiography Cohort and the Remaining MGBB Participants

Baseline Characteristics	Study Participants (n=3,518)	Remaining MGB Biobank Participants (n=49,607)	P
**Female, n (%)**	1,051 (29.9)	28,498 (57.4)	<.001
**Age at genotyping, median (IQR)**	68.10 (60.20-75.20)	55.10 (38.65-66.40)	<.001
**Race**			
White, n (%)	3,152 (89.6)	41,779 (84.2)	<.001
Black, n (%)	135 (3.8)	2,461 (5.0)	
Asian, n (%)	38 (1.1)	1,443 (2.9)	
Other, n (%)	120 (3.4)	3,924 (7.9)	
NA, n (%)	73 (2.1)	0 (0)	
**Comorbidities**[a]			
Hypertension, n (%)	3,455 (98.5)	38,691 (80.0)	<.001
Hypercholesterolemia, n (%)	2,724 (77.7)	12,409 (25.7)	<.001
Diabetes mellitus type 2, n (%)	1,586 (45.2)	7,849 (16.2)	<.001
Current smoking, n (%)	350 (10.0)	3,946 (8.2)	<.001
**Genomic driver**			
FH variant, n (%)[b]	26 (0.8)	253 (0.5)	0.09
High CAD PRS, n (%)	1,191 (33.8)	9,424 (19.0)	<.001
CHIP variant, n (%)[c]	466 (20.8)	5,181 (10.5)	<.001

Abbreviations: BMI, body mass index; CAD, coronary artery disease; CHIP, clonal hematopoiesis of indeterminate potential; FH, familial hypercholesterolemia; MGB, Mass General Brigham; NA, not available; PRS, polygenic risk score.

[a] Comorbidities were curated with the index date defined as the biospecimen collection date, among genotyped Mass General Brigham Biobank. Data were available for 51,862 individuals, including 3,507 patients who were part of our study cohort.

[b] The proportion of people with FH variant out of individuals with available whole exome sequencing data used for FH analysis (n=3,433).

[c] The proportion of people with CHIP after filtering the CHIP ascertainment date within the range of 10 years before and 1 year after the initial angiogram (n=2,239).

© 2025 Supriami K et al. *JAMA Network Open.*

eTable 3. Angiographic Characteristics and Outcomes Based on the Presence of Any Genomic Drivers

Angiographic Characteristics and Outcomes	Any genomic driver[a] (n=1,509)	No genomic driver (n=2,009)	*P-value*
**Age at coronary angiography**, median (IQR)	64.0 (55.0-71.0)	64.0 (55.0-72.0)	0.96
**Initial presentation**			
STEMI, n (%)	93 (6.2)	142 (7.1)	<.001
NSTEMI, n (%)	226 (15.0)	212 (10.6)	
Unstable Angina, n (%)	138 (9.1)	155 (7.7)	
Stable CAD/ischemia, n (%)	764 (50.6)	883 (44.0)	
Other, n (%)	288 (19.1)	617 (30.6)	
**CAD severity**			
No CAD, n (%)	263 (17.5)	687 (34.2)	<.001
Mild CAD, n (%)	128 (8.5)	212 (10.5)	
Moderate CAD, n (%)	150 (9.9)	196 (9.8)	
Severe CAD, n (%)	968 (64.1)	914 (45.5)	
**CAD burden[b]**			
Non-obstructive, n (%)	278 (22.3)	408 (30.9)	<.001
1-vessel disease, n (%)	441 (35.4)	459 (34.7)	
2-vessel disease, n (%)	245 (19.7)	220 (16.6)	
3-vessel disease, n (%)	160 (12.8)	123 (9.3)	
Left main disease, n (%)	122 (9.8)	112 (8.5)	
**Gensini score**, median (IQR)	28.0 (4.0-64.0)	8.0 (0-41.0)	<.001
**Outcome**			
Repeat angiogram, n (%)	653 (43.3)	633 (31.5)	<.001
Number of angiograms, mean (SD)	2.14 (2.27)	1.74 (1.83)	<.001
Revascularization, n (%)[c]	355 (23.6)	304 (15.2)	<.001
In-stent restenosis, n (%)[d]	168 (11.2)	118 (5.9)	<.001

© 2025 Supriami K et al. *JAMA Network Open.*

**Angiographic Characteristics and Outcomes**	**Any genomic driver**[a] (n=1,509)	**No genomic driver** (n=2,009)	***P-value***
Heart failure, n (%)[e]	112 (16.1)	128 (14.1)	0.30
All-cause mortality, n (%)[f]	407 (27.0)	478 (23.9)	0.04

Abbreviations: CAD, coronary artery disease; NSTEMI, non-ST-elevation myocardial infarction; PRS, polygenic risk score; STEMI, ST-elevation myocardial infarction.

[a] Any genomic driver indicated the presence of at least one of the following: familial hypercholesterolemia, high polygenic risk score, and/or clonal hematopoiesis of indeterminate potential.

[b] The proportion of CAD burden was calculated by excluding those who presented without CAD, leaving 2,568 individuals in the analysis with 1,246 among them having at least 1 genomic driver of CAD.

[c] The denominator of revascularization for any genomic driver and no genomic driver groups are 1,502 and 2,004, respectively.

[d] The denominator of in-stent restenosis for any genomic driver and no genomic driver groups are 1,494 and 1,997, respectively.

[e] The denominator of heart failure for any genomic driver and no genomic driver groups are 696 and 907, respectively.

[f] The denominator of all-cause mortality for any genomic driver and no genomic driver groups are 1,505 and 2,000, respectively.

© 2025 Supriami K et al. *JAMA Network Open.*

eTable 4. Angiographic Characteristics and Outcomes by Familial Hypercholesterolemia (FH) Variant Status

**Angiographic Characteristics and Outcomes**	**FH variant carrier** (n=26)	**Non-FH variant carrier** (n=3,407)	***P-value***
**Age at coronary angiography**, median (IQR)	62.0 (52.8- 68.0)	64.0 (55.0-72.0)	0.38
**Initial presentation**			
STEMI, n (%)	1 (3.8)	230 (6.8)	0.01
NSTEMI, n (%)	9 (34.6)	419 (12.3)	
Unstable Angina, n (%)	1 (3.8)	281 (8.2)	
Stable CAD/ischemia, n (%)	11 (42.3)	1,596 (46.8)	
Other, n (%)	4 (15.5)	881 (25.9)	
**CAD severity**			
No CAD, n (%)	4 (15.4)	926 (27.2)	0.32
Mild CAD, n (%)	1 (3.8)	327 (9.6)	
Moderate CAD, n (%)	3 (11.5)	334 (9.8)	
Severe CAD, n (%)	18 (69.3)	1,820 (53.4)	
**CAD burden**[a]			
Non-obstructive, n (%)	4 (18.2)	661 (26.6)	0.57
1-vessel disease, n (%)	8 (36.4)	869 (35.0)	
2-vessel disease, n (%)	6 (27.3)	451 (18.2)	
3-vessel disease, n (%)	1 (4.5)	274 (11.0)	
Left main disease, n (%)	3 (13.6)	226 (9.2)	
**Gensini score**, median (IQR)	31.0 (10.0-54.3)	16.0 (0-50.5)	0.60
**Outcome**			
Repeat angiogram, n (%)	15 (57.7)	1,231 (36.1)	0.04
Number of angiograms, mean (SD)	2.04 (1.11)	1.91 (2.06)	0.76
Revascularization, n (%)[b]	9 (34.6)	631 (18.6)	0.07
In-stent restenosis, n (%)[c]	4 (16.0)	272 (8.0)	0.28
Heart failure, n (%)[d]	0 (0)	231 (14.9)	0.57
All-cause mortality, n (%)[e]	8 (30.8)	857 (25.3)	0.68

Abbreviations: CAD, coronary artery disease; FH, familial hypercholesterolemia; NA, not applicable; NSTEMI, non-ST-elevation myocardial infarction; PRS, polygenic risk score; STEMI, ST-elevation myocardial infarction.

© 2025 Supriami K et al. *JAMA Network Open.*

[a] The proportion of CAD burden was calculated by excluding those who presented without CAD, leaving 2,503 individuals in the analysis with 22 among them being FH variant carriers.
[b] The denominator of revascularization for FH variant and non-FH variant groups are 26 and 3,395, respectively.
[c] The denominator of in-stent restenosis for FH variant and non-FH variant groups are 25 and 3,381, respectively.
[d] The denominator of heart failure for FH variant and non-FH variant groups are 7 and 1,551, respectively.
[e] The denominator of all-cause mortality for FH variant and non-FH variant groups are 26 and 3,394, respectively.

© 2025 Supriami K et al. *JAMA Network Open.*

eTable 5. Angiographic Characteristics and Outcomes by Coronary Artery Disease (CAD) Polygenic Risk Score (PRS) Group

Angiographic Characteristics and Outcomes	High PRS group (n=1,191)	Non-high PRS group (n=2,327)	*P-value*
**Age at coronary angiography**, median (IQR)	63.0 (54.0-70.0)	65.0 (56.0-73.0)	<.01
**Initial presentation**			
STEMI, n (%)	80 (6.7)	155 (6.7)	<.001
NSTEMI, n (%)	186 (15.6)	252 (10.8)	
Unstable Angina, n (%)	112 (9.4)	181 (7.8)	
Stable CAD/ischemia, n (%)	612 (51.4)	1,035 (44.5)	
Other, n (%)	201 (16.9)	704 (30.2)	
**CAD severity**			
No CAD, n (%)	183 (15.4)	767 (33.0)	<.001
Mild CAD, n (%)	87 (7.3)	253 (10.9)	
Moderate CAD, n (%)	116 (9.7)	230 (9.9)	
Severe CAD, n (%)	805 (67.6)	1,077 (46.2)	
**CAD burden**[a]			
Non-obstructive, n (%)	203 (20.1)	483 (31.0)	<.001
1-vessel disease, n (%)	362 (35.9)	538 (34.5)	
2-vessel disease, n (%)	203 (20.1)	262 (16.8)	
3-vessel disease, n (%)	141 (14.0)	142 (9.1)	
Left main disease, n (%)	99 (9.9)	135 (8.6)	
**Gensini score**, median (IQR)	32.0 (7.0-68.0)	9.0 (0-42.0)	<.001
**Outcome**			
Repeat angiogram, n (%)	539 (45.3)	747 (32.1)	<.001
Number of angiograms, mean (SD)	2.26 (2.45)	1.74 (1.77)	<.001
Revascularization, n (%)[b]	296 (25.0)	363 (15.6)	<.001
In-stent restenosis, n (%)[c]	152 (12.9)	134 (5.8)	<.001
Heart failure, n (%)[d]	81 (15.1)	159 (14.9)	1.00
All-cause mortality, n (%)[e]	301 (25.4)	548 (25.2)	0.95

Abbreviations: CAD, coronary artery disease; NSTEMI, non-ST-elevation myocardial infarction; PRS, polygenic risk score; STEMI, ST-elevation myocardial infarction.

[a] The proportion of CAD burden was calculated by excluding those who presented without CAD, leaving 2,568 individuals in the analysis with 1,008 among them in the high PRS CAD group.

© 2025 Supriami K et al. *JAMA Network Open.*

[b] The denominator of revascularization for high PRS and non-high PRS groups are 1,184 and 2,322, respectively.
[c] The denominator of in-stent restenosis for high PRS and non-high PRS groups are 1,179 and 2,312, respectively.
[d] The denominator of heart failure for high PRS and non-high PRS groups are 538 and 1,065, respectively.
[e] The denominator of all-cause mortality for high PRS and non-high PRS groups are 1,187 and 2,318, respectively.

© 2025 Supriami K et al. *JAMA Network Open.*

eTable 6. Angiographic Characteristics and Outcomes by Clonal Hematopoiesis of Indeterminate Potential (CHIP) Mutation

Angiographic Characteristics and Outcomes	Had CHIP (n=466)	No CHIP (n=1,773)	*P-value*
**Age at coronary angiography**, median (IQR)	69.0 (62.0-75.8)	62.0 (54.0-70.0)	<.001
**Initial presentation**			
STEMI, n (%)	20 (4.3)	129 (7.3)	0.11
NSTEMI, n (%)	65 (13.9)	227 (12.8)	
Unstable Angina, n (%)	36 (7.7)	137 (7.7)	
Stable CAD/ischemia, n (%)	231 (49.6)	804 (45.3)	
Other, n (%)	114 (24.5)	476 (26.9)	
**CAD severity**			
No CAD, n (%)	95 (20.4)	527 (29.7)	0.001
Mild CAD, n (%)	56 (12.0)	163 (9.2)	
Moderate CAD, n (%)	46 (9.9)	161 (9.1)	
Severe CAD, n (%)	269 (57.7)	922 (52.0)	
**CAD burden**[a]			
Non-obstructive, n (%)	102 (27.5)	324 (26.0)	0.62
1-vessel disease, n (%)	117 (31.5)	436 (35.0)	
2-vessel disease, n (%)	68 (18.4)	229 (18.4)	
3-vessel disease, n (%)	42 (11.3)	143 (11.5)	
Left main disease, n (%)	42 (11.3)	114 (9.1)	
**Gensini score**, median (IQR)	20.0 (2.5-56.8)	15.0 (0-50)	0.02
**Outcome**			
Repeat angiogram, n (%)	182 (39.1)	617 (34.8)	0.10
Number of angiograms, mean (SD)	1.80 (1.49)	1.77 (1.62)	0.70
Revascularization, n (%))[b]	97 (20.8)	294 (16.7)	0.04
In-stent restenosis, n (%)[c]	37 (8.0)	114 (6.5)	0.30
Heart failure, n (%)[d]	54 (21.9)	159 (16.0)	0.04
All-cause mortality, n (%)[e]	172 (37.0)	429 (24.3)	<.001

Abbreviations: CAD, coronary artery disease; CHIP, clonal hematopoiesis of indeterminate potential; NSTEMI, non-ST-elevation myocardial infarction; PRS, polygenic risk score; STEMI, ST-elevation myocardial infarction.

[a] The proportion of CAD burden was calculated by excluding those who presented without CAD, leaving 1,617 individuals in the analysis with 371 among them having CHIP.

© 2025 Supriami K et al. *JAMA Network Open.*

[b] The denominator of revascularization for had CHIP and no CHIP groups are 466 and 1,765, respectively.
[c] The denominator of in-stent restenosis for had CHIP and no CHIP groups are 463 and 1,760, respectively.
[d] The denominator of heart failure for had CHIP and no CHIP groups are 247 and 995, respectively.
[e] The denominator of all-cause mortality for had CHIP and no CHIP groups are 465 and 1,767, respectively.

© 2025 Supriami K et al. *JAMA Network Open.*

eTable 7. List of Pathogenic or Likely Pathogenic Variants of Familial Hypercholesterolemia in the Study Cohort (N = 26)

Variant	Gene (Variant Type)	Amino acid or cDNA change	Number of carriers
chr1:55039940:G:T	PCSK9 Missense	p.Asp35Tyr	1
chr19:11100255:T:G	LDLR Missense	p.Cys34Gly	1
chr19:11102705:C:T	LDLR Missense	p.Arg78Cys	1
chr19:11102714:C:T	LDLR Missense	p.Arg81Cys	1
chr19:11102774:G:A	LDLR Missense	p.Glu101Lys	1
chr19:11105258:G:T	LDLR Missense	p.Asp118Tyr	1
chr19:11105414:G:A	LDLR Frameshift	p.Asp170fs	1
chr19:11105430:A:T	LDLR Missense	p.Asp175Val	1
chr19:11105587:C:G	LDLR Deletion	p.Glu228_Cys231del	1
chr19:11110714:G:A	LDLR Missense	p.Gly335Ser	1
chr19:11110715:G:A	LDLR Missense	p.Gly335Asp	1
chr19:11110730:G:A	LDLR Missense	p.Cys340Tyr	1
chr19:11113286:G:A	LDLR Missense	p.Ala399Ser	1
chr19:11113620:G:A	LDLR Missense	p.Asp482Asn	1
chr19:11113752:C:T	LDLR Missense	p.Pro526Ser	1
chr19:11116153:G:A	LDLR Frameshift	p.Gly549fs	1
chr19:11116197:A:C	LDLR Missense	p.Asn564Asp	1
chr19:11120423:T:G	LDLR Missense	p.Cys681Gly	1
chr19:11120425:C:A	LDLR Missense	p.Cys681Trp	1
chr19:11129653:G:A	LDLR Missense	p.Gly844Ser	1
chr2:21006288:C:T	APOB Missense	p.Arg3527Leu	6

© 2025 Supriami K et al. *JAMA Network Open.*

eTable 8. Frequency of Clonal Hematopoiesis of Indeterminate Potential Driver Genes in the Study Cohort (N = 466)

Gene	Frequency	Rank
DNMT3A	181	1
TET2	115	2
ASXL1	66	3
PPM1D	48	4
SF3B1	22	5
SRSF2	16	6
TP53	14	7
PRPF8	8	8
JAK2, *ZNF318*	7	9
PHIP, *U2AF1*	6	10
GNB1, *YLPM1*, *ZBTB33*	5	11
BCOR, *CBL*, *CUX1*, *IDH1*, *NXF1*, *ZRSR2*	3	12
ASXL2, *BCORL1*, *IDH2*, *KDM6A*, *MPL*, *PDS5B*, *STAG1*	2	13
CALR, *EP300*, *ETV6*, *EZH2*, *KRAS*, *RUNX1*, *SRCAP*, *STAG2*, *SUZ12*	1	14

© 2025 Supriami K et al. *JAMA Network Open.*

eTable 9. Univariable and Multivariable-adjusted Associations of Coronary Angiography Characteristics with the Presence of Genomic Drivers

Characteristics	Cases / Total N	Model 1			Model 2			Model 3		
		OR	95% CI	*P*	aOR	95% CI	*P*	aOR	95% CI	*P*
**Severity CAD**										
No CAD	263/1,509	*Reference*								
Mild	128/1,509	1.58	1.22-2.05	6.23×10^{-4}	1.61	1.24-2.10	4.09×10^{-4}	1.56	1.19-2.04	1.10×10^{-3}
Moderate	150/1,509	2.00	1.55-2.58	1.09×10^{-7}	2.15	1.65-2.79	1.20×10^{-8}	2.09	1.60-2.72	5.67×10^{-8}
Severe	968/1,509	2.77	2.34-3.27	2.57×10^{-32}	2.94	2.47-3.52	8.57×10^{-33}	2.83	2.36-3.39	2.17×10^{-29}
**Burden CAD**										
Non-obstructive CAD	278/1,246	*Reference*								
Single vessel	441/1,246	1.41	1.15-1.72	7.96×10^{-4}	1.39	1.13-1.70	1.54×10^{-3}	1.38	1.13-1.69	1.96×10^{-3}
Multi-vessel	405/1,246	1.73	1.41-2.14	2.72×10^{-7}	1.77	1.43-2.19	1.61×10^{-7}	1.75	1.41-2.17	3.39×10^{-7}
Left main	122/1,246	1.60	1.19-2.15	2.06×10^{-3}	1.77	1.31-2.40	2.21×10^{-4}	1.78	1.31-2.41	2.15×10^{-4}
**Initial presentation**										
No CAD	263/1,509	*Reference*								
Stable CAD	825/1,509	2.40	2.02-2.84	1.01×10^{-23}	2.54	2.12-3.04	3.84×10^{-24}	2.45	2.04-2.94	1.36×10^{-21}
ACS	421/1,509	2.60	2.14-3.16	1.22×10^{-21}	2.67	2.19-3.26	7.57×10^{-22}	2.57	2.09-3.15	1.33×10^{-19}

Genomic driver	Changes of Gensini score					
	Model 1		Model 2		Model 3	
	Estimate (95% CI)	*P*	Estimate (95% CI)	*P*	Estimate (95% CI)	*P*
No genomic driver	*Reference*					
Any genomic driver	18.21 (14.85-21.57)	$<2 \times 10^{-6}$	18.11 (14.85-21.38)	$<2 \times 10^{-6}$	17.47 (14.21-20.73)	$<2 \times 10^{-6}$

Abbreviations: ACS, acute coronary syndromes; aOR, adjusted odds ratios; CAD, coronary artery disease.
Odd ratios (OR) with corresponding 95% confidence intervals (CI) were calculated using multinomial logistic regression models for categorical dependent variables. Model 1 was the crude, unadjusted analysis; model 2 included age at the time of coronary angiography, sex, and the first 4 principal components of genetic ancestry, as covariates; and model 3 was adjusted for age at the time of coronary angiography, sex, the first 4 principal components of genetic ancestry, hypertension, hypercholesterolemia, diabetes mellitus type 2, and current smoking. Multi-vessel was defined as a composite of 2-vessel or 3-vessel coronary lesions. ACS was defined as either ST-elevation myocardial infarction, non-ST-elevation myocardial infarction, or unstable angina. Patients with initial presentation of stable CAD or ACS but without any angiographic evidence of CAD was reclassified into the no CAD group (reference).

© 2025 Supriami K et al. *JAMA Network Open.*

eTable 10. Univariable and Multivariable-adjusted Associations of Coronary Angiography Characteristics with Familial Hypercholesterolemia (FH) Variant Status

Characteristics	Cases / Total N	Model 1			Model 2			Model 3		
		OR	95% CI	*P*	aOR	95% CI	*P*	aOR	95% CI	*P*
**Severity CAD**										
No / mild	5/26	*Reference*								
Moderate / severe	21/26	2.44	0.99-7.33	0.07	3.01	1.18-9.28	0.03	2.94	1.14-9.12	0.04
**Severity CAD**										
No CAD	4/26	*Reference*								
Mild	1/26	0.71	0.08-6.36	0.76	0.84	0.09-7.64	0.88	0.83	0.09-7.57	0.87
Moderate	3/26	2.08	0.46-9.34	0.34	2.70	0.58-12.55	0.20	2.74	0.58-12.89	0.20
Severe	18/26	2.29	0.77-6.78	0.14	2.97	0.96-9.16	0.06	2.88	0.92-9.06	0.07
**Burden CAD**										
Non-obstructive CAD	4/22	*Reference*								
Single vessel	8/22	1.52	0.46-5.07	0.49	1.53	0.45-5.13	0.50	1.49	0.44-5.03	0.52
Multi-vessel	7/22	1.60	0.46-5.47	0.46	1.77	0.51-6.19	0.37	1.66	0.47-5.84	0.43
Left main	3/22	2.19	0.49-9.88	0.31	2.67	0.58-12.35	0.21	2.62	0.56-12.14	0.22
**Initial presentation**										
No CAD	4/26	*Reference*								
Stable CAD	11/26	1.66	0.54-5.17	0.38	2.10	0.64-6.83	0.22	2.02	0.61-6.70	0.25
ACS	11/26	2.84	0.89-9.10	0.08	3.31	1.01-10.78	0.05	3.24	0.98-10.70	0.05

Genomic driver	Changes of Gensini score					
	Model 1		Model 2		Model 3	
	Estimate (95% CI)	*P*	Estimate (95% CI)	*P*	Estimate (95% CI)	*P*
Non-FH carrier	*Reference*					
FH variant carrier	5.28 (-14.47-25.04)	0.60	7.49 (-11.66-26.65)	0.44	6.53 (-12.53-25.58)	0.50

Abbreviations: ACS, acute coronary syndromes; aOR, adjusted odds ratios; CAD, coronary artery disease.
Odd ratios (OR) with corresponding 95% confidence intervals (CI) were calculated using multinomial logistic regression models for categorical dependent variables and binomial logistic regression models for binary dependent variables. Model 1 was the crude, unadjusted analysis; model 2 included age at the time of coronary angiography, sex, and the first 4 principal components of genetic ancestry, as covariates; and model 3 was adjusted for age at the time of coronary angiography, sex, the first 4 principal components of genetic ancestry, hypertension, hypercholesterolemia, diabetes mellitus type 2, and current smoking. Multi-vessel was defined as a composite of 2-vessel or 3-vessel coronary lesions. ACS was defined as either ST-elevation myocardial infarction, non-ST-elevation myocardial infarction, or unstable angina. Patients with initial presentation of stable CAD or ACS but without any angiographic evidence of CAD was reclassified into the no CAD group (reference).

© 2025 Supriami K et al. *JAMA Network Open.*

eTable 11. Age and Sex-matched Univariable and Multivariable-adjusted Associations of Coronary Angiography Characteristics with Familial Hypercholesterolemia (FH) Variant Status

Characteristics	Cases / Total N	Model 1			Model 2			Model 3		
		OR	95% CI	*P*	aOR	95% CI	*P*	aOR	95% CI	*P*
Severity CAD										
No / mild	5/26	*Reference*								
Moderate / severe	21/26	4.54	1.70-14.41	4.70×10^{-3}	7.42	2.33-28.6	1.52×10^{-3}	7.47	2.12-32.13	3.35×10^{-3}
Severity CAD										
No CAD	4/26	*Reference*								
Mild	1/26	1.25	0.12-12.53	0.85	0.95	0.08-11.12	0.97	0.98	0.08-11.66	0.99
Moderate	3/26	3.37	0.65-17.51	0.15	5.60	0.93-33.71	0.06	5.27	0.80-34.87	0.08
Severe	18/26	5.06	1.58-16.22	0.01	7.89	2.08-29.86	2.36×10^{-3}	8.46	1.98-36.16	3.95×10^{-3}
Burden CAD										
Non-obstructive CAD	4/22	*Reference*								
Single vessel	8/22	1.65	0.43-6.34	0.46	2.11	0.45-9.96	0.34	2.50	0.49-12.78	0.27
Multi-vessel	7/22	2.37	0.58-9.72	0.23	3.58	0.75-17.04	0.11	3.01	0.59-15.33	0.19
Left main	3/22	4.75	0.69-32.71	0.11	4.25	0.52-34.52	0.18	2.69	0.27-26.75	0.40
Initial presentation										
No CAD	4/26	*Reference*								
Stable CAD	12/26	3.55	1.06-11.93	0.04	4.68	1.26-17.41	0.02	3.74	0.91-15.37	0.07
ACS	10/26	5.36	1.50-19.07	0.01	6.44	1.68-24.71	0.01	6.11	1.52-24.55	0.01

Genomic driver	Changes of Gensini score					
	Model 1		Model 2		Model 3	
	Estimate (95% CI)	*P*	Estimate (95% CI)	*P*	Estimate (95% CI)	*P*
Non-FH carrier	*Reference*					
FH variant carrier	18.54 (0.41-36.67)	0.05	17.76 (-0.60-34.92)	0.06	15.71 (-2.16-33.58)	0.09

Abbreviations: ACS, acute coronary syndromes; aOR, adjusted odds ratios; CAD, coronary artery disease.
Odd ratios (OR) with corresponding 95% confidence intervals (CI) were calculated using multinomial logistic regression models for categorical dependent variables and binomial logistic regression models for binary dependent variables. Model 1 was the crude, unadjusted analysis; model 2 included age at the time of coronary angiography, sex, and the first 4 principal components of genetic ancestry, as covariates; and model 3 was adjusted for age at the time of coronary angiography, sex, the first 4 principal components of genetic ancestry, hypertension, hypercholesterolemia, diabetes mellitus type 2, and current smoking. A 1:4 propensity score matching for age and sex resulted in a sample size of 130 participants (26 in the FH carrier group and 104 in the control non-FH carrier group). Multi-vessel was defined as a composite of 2-vessel or 3-vessel coronary lesions. ACS was defined as either ST-elevation myocardial infarction, non-ST-elevation myocardial infarction, or unstable angina. Patients with initial presentation of stable CAD or ACS but without any angiographic evidence of CAD was reclassified into the no CAD group (reference).

© 2025 Supriami K et al. *JAMA Network Open.*

eTable 12. Univariable and Multivariable-adjusted Associations of Coronary Angiography Characteristics with Coronary Artery Disease (CAD) Polygenic Risk Score (PRS)

Characteristics	Cases / Total N	Model 1			Model 2			Model 3		
		OR	95%CI	*P*	aOR	95%CI	*P*	aOR	95%CI	*P*
Severity CAD										
No CAD	183/1,191	*Reference*								
Mild	87/1,191	1.44	1.08-1.93	$1.44x10^{-2}$	1.65	1.22-2.22	$1.07x10^{-3}$	1.57	1.16-2.12	$3.29x10^{-3}$
Moderate	116/1,191	2.11	1.60-2.78	$9.98x10^{-8}$	2.68	2.01-3.56	$1.52x10^{-11}$	2.56	1.92-3.41	$1.77x10^{-10}$
Severe	805/1,191	3.13	2.60-3.77	$1.45x10^{-33}$	3.81	3.13-4.65	$3.05x10^{-40}$	3.61	2.95-4.42	$7.84x10^{-36}$
Burden CAD										
Non-obstructive CAD	203/1,008	*Reference*								
Single vessel	362/1,008	1.60	1.30-1.98	$1.26x10^{-5}$	1.54	1.24-1.91	$8.69x10^{-5}$	1.53	1.23-1.90	$1.08x10^{-4}$
Multi-vessel	344/1,008	2.03	1.63-2.52	$2.21x10^{-10}$	2.08	1.66-2.61	$1.49x10^{-10}$	2.06	1.65-2.58	$2.83x10^{-10}$
Left main	99/1,008	1.74	1.28-2.37	$3.77x10^{-4}$	2.06	1.50-2.82	$7.85x10^{-6}$	2.05	1.49-2.81	$8.64x10^{-6}$
Initial presentation										
No CAD	183/1,191	*Reference*								
Stable CAD	653/1,191	2.56	2.12-3.09	$1.61x10^{-22}$	3.14	2.57-3.83	$5.75x10^{-29}$	2.97	2.42-3.65	$1.39x10^{-25}$
ACS	355/1,191	3.04	2.46-3.76	$5.83x10^{-25}$	3.41	2.74-4.24	$3.62x10^{-28}$	3.23	2.59-4.04	$4.31x10^{-25}$

Genomic driver	Changes in Gensini Score					
	Model 1		Model 2		Model 3	
	Estimate (95% CI)	*P*	Estimate (95% CI)	*P*	Estimate (95% CI)	*P*
Standardized adjusted CAD PRS	11.76 (10.15-13.37)	$<2x10^{-6}$	12.51 (10.94-14.07)	$<2x10^{-6}$	12.07 (10.50-13.64)	$<2x10^{-6}$

Abbreviations: ACS, acute coronary syndromes; aOR, adjusted odds ratios; CAD, coronary artery disease.
Odd ratios (OR) with corresponding 95% confidence intervals (CI) were calculated using multinomial logistic regression models for categorical dependent variables. Model 1 was the crude, unadjusted analysis; model 2 included age at the time of coronary angiography, sex, and the first 4 principal components of genetic ancestry, as covariates; and model 3 was adjusted for age at the time of coronary angiography, sex, the first 4 principal components of genetic ancestry, hypertension, hypercholesterolemia, diabetes mellitus type 2, and current smoking. Multi-vessel was defined as a composite of 2-vessel or 3-vessel coronary lesions. ACS was defined as either ST-elevation myocardial infarction, non-ST-elevation myocardial infarction, or unstable angina. Patients with initial presentation of stable CAD or ACS but without any angiographic evidence of CAD was reclassified into the no CAD group (reference).

© 2025 Supriami K et al. *JAMA Network Open.*

eTable 13. Univariable and Multivariable-adjusted Associations of Coronary Angiography Characteristics with Coronary Artery Disease (CAD) Polygenic Risk Score (PRS) in the Replication Cohort (N=783)

Characteristics	Cases / Total N	Model 1			Model 2			Model 3		
		OR	95%CI	*P*	aOR	95%CI	*P*	aOR	95%CI	*P*
Severity CAD										
No CAD	42/256	*Reference*								
Mild	17/256	1.33	0.70-2.52	0.39	1.71	0.87-3.35	0.12	1.62	0.82-3.20	0.16
Moderate	33/256	2.58	1.49-4.47	7.35×10^{-4}	3.24	1.82-5.77	6.66×10^{-5}	3.15	1.76-5.65	1.13×10^{-4}
Severe	164/256	2.75	1.86-4.06	3.84×10^{-7}	3.75	2.44-5.77	1.93×10^{-9}	3.61	2.33-5.58	7.87×10^{-9}
Burden CAD										
Non-obstructive CAD	50/214	*Reference*								
Single vessel	61/214	1.06	0.67-1.67	0.80	1.13	0.70-1.81	0.62	1.12	0.70-1.81	0.64
Multi-vessel	78/214	1.86	1.18-2.92	0.01	2.03	1.26-3.28	3.70×10^{-3}	2.02	1.25-3.27	4.16×10^{-3}
Left main	25/214	1.47	0.80-2.71	0.21	1.85	0.97-3.51	0.06	1.89	0.99-3.61	0.05
Initial presentation										
No CAD	42/256	*Reference*								
Stable CAD	151/256	2.35	1.59-3.48	1.88×10^{-5}	3.17	2.07-4.87	1.33×10^{-7}	3.05	1.98-4.71	4.24×10^{-7}
ACS	63/256	3.00	1.88-4.79	4.14×10^{-6}	3.68	2.24-6.04	2.64×10^{-7}	3.56	2.16-5.89	7.04×10^{-7}

Genomic driver	Changes in Gensini Score					
	Model 1		Model 2		Model 3	
	Estimate (95% CI)	*P*	Estimate (95% CI)	*P*	Estimate (95% CI)	*P*
Standardized adjusted CAD PRS	11.76 (10.15-13.37)	4.54×10^{-14}	12.51 (10.94-14.07)	2.95×10^{-15}	12.07 (10.50-13.64)	7.72×10^{-15}

Abbreviations: ACS, acute coronary syndromes; aOR, adjusted odds ratios; CAD, coronary artery disease.
Odd ratios (OR) with corresponding 95% confidence intervals (CI) were calculated using multinomial logistic regression models for categorical dependent variables. Model 1 was the crude, unadjusted analysis; model 2 included age at the time of coronary angiography, sex, and the first 4 principal components of genetic ancestry, as covariates; and model 3 was adjusted for age at the time of coronary angiography, sex, the first 4 principal components of genetic ancestry, hypertension, hypercholesterolemia, diabetes mellitus type 2, and current smoking. Multi-vessel was defined as a composite of 2-vessel or 3-vessel coronary lesions. ACS was defined as either ST-elevation myocardial infarction, non-ST-elevation myocardial infarction, or unstable angina. Patients with initial presentation of stable CAD or ACS but without any angiographic evidence of CAD was reclassified into the no CAD group (reference).

© 2025 Supriami K et al. *JAMA Network Open.*

eTable 14. Univariable and Multivariable-adjusted Associations of Coronary Angiography Characteristics with Clonal Hematopoiesis of Indeterminate Potential (CHIP) Mutation

Characteristics	Cases / Total N	Model 1			Model 2			Model 3		
		OR	95%CI	*P*	aOR	95%CI	*P*	aOR	95%CI	*P*
**Severity CAD**										
No CAD	95/466	*Reference*								
Mild, moderate, or severe	371/466	1.65	1.29-2.12	$6.98x10^{-5}$	1.22	0.94-1.59	0.13	1.24	0.95-1.62	0.12
**Severity CAD**										
No CAD	95/466	*Reference*								
Mild	56/466	1.91	1.31-2.77	$7.25x10^{-4}$	1.45	0.98-2.14	0.06	1.46	0.99-2.16	0.06
Moderate	46/466	1.58	1.07-2.35	0.02	1.12	0.74-1.69	0.60	1.13	0.75-1.72	0.56
Severe	269/466	1.62	1.25-2.09	$2.43x10^{-4}$	1.20	0.91-1.57	0.20	1.21	0.91-1.60	0.19
**Burden CAD**										
Non-obstructive and obstructive CAD, except LM	329/371	*Reference*								
Left-main	42/371	1.27	0.86-1.83	0.21	1.09	0.73-1.59	0.67	1.11	0.75-1.63	0.59
**Burden CAD**										
Non-obstructive CAD	102/371	*Reference*								
Single vessel	117/371	0.85	0.63-1.15	0.30	0.90	0.66-1.24	0.53	0.90	0.65-1.23	0.50
Multi-vessel	110/371	0.94	0.69-1.28	0.69	0.92	0.67-1.27	0.61	0.91	0.66-1.26	0.58
Left main	42/371	1.17	0.77-1.78	0.46	1.02	0.66-1.57	0.93	1.04	0.67-1.61	0.86
**Initial presentation**										
No CAD	95/466	*Reference*								
Stable CAD	264/466	1.79	1.38-2.32	$1.07x10^{-5}$	1.25	0.95-1.64	0.11	1.26	0.95-1.67	0.11
ACS	107/466	1.39	1.03-1.88	0.03	1.16	0.85-1.60	0.35	1.19	0.86-1.64	0.30

Genomic driver	Changes of Gensini score					
	Model 1		Model 2		Model 3	
	Estimate (95% CI)	*P*	Estimate (95% CI)	*P*	Estimate (95% CI)	*P*
No CHIP	*Reference*					
Had CHIP	6.42 (0.99-11.85)	0.02	0.82 (-4.64-6.27)	0.77	0.82 (-4.59-6.23)	0.77

Abbreviations: ACS, acute coronary syndromes; aOR, adjusted odds ratios; CAD, coronary artery disease.
Odd ratios (OR) with corresponding 95% confidence intervals (CI) in multivariable analyses were calculated using multinomial logistic regression models for categorical dependent variables and binomial logistic regression models for binary dependent variables. Model 1 was the crude, unadjusted analysis; model 2 included age at the time of coronary angiography, sex, and the first 4 principal components of genetic

© 2025 Supriami K et al. *JAMA Network Open.*

ancestry, as covariates; and model 3 was adjusted for age at the time of coronary angiography, sex, the first 4 principal components of genetic ancestry, hypertension, hypercholesterolemia, diabetes mellitus type 2, and current smoking. Multi-vessel was defined as a composite of 2-vessel or 3-vessel coronary lesions. ACS was defined as either ST-elevation myocardial infarction, non-ST-elevation myocardial infarction, or unstable angina. Patients with initial presentation of stable CAD or ACS but without any angiographic evidence of CAD was reclassified into the no CAD group (reference).

© 2025 Supriami K et al. *JAMA Network Open.*

eTable 15. Univariable and Multivariable-adjusted Associations of Coronary Angiography Characteristics with Clonal Hematopoiesis of Indeterminate Potential (CHIP) Mutation by Driver Genes and Clone Size

A. *Non-DNMT3A vs no CHIP (reference)*

Characteristics	Cases / Total N	Model 1			Model 2			Model 3		
		OR	95%CI	*P*	aOR	95%CI	*P*	aOR	95%CI	*P*
**Severity CAD**										
No CAD	52/295	*Reference*								
Mild	30/295	1.87	1.15-3.02	0.01	1.31	0.79-2.15	0.29	1.30	0.79-2.15	0.31
Moderate	28/295	1.76	1.08-2.88	0.02	1.16	0.70-1.94	0.56	1.16	0.69-1.93	0.58
Severe	185/295	2.03	1.47-2.82	1.95×10^{-5}	1.42	1.01-2.00	0.04	1.41	0.99-2.00	0.05
**Burden CAD**										
Non-obstructive CAD	58/243	*Reference*								
Single vessel	81/243	1.04	0.72-1.50	0.84	1.14	0.78-1.67	0.49	1.14	0.78-1.67	0.50
Multi-vessel	76/243	1.14	0.79-1.66	0.49	1.12	0.78-1.66	0.55	1.11	0.75-1.64	0.60
Left main	28/243	1.37	0.83-2.26	0.21	1.19	0.71-1.99	0.51	1.20	0.72-2.02	0.48
**Initial presentation**										
No CAD	52/295	*Reference*								
Stable CAD	174/295	2.15	1.55-2.99	4.75×10^{-6}	1.39	0.98-1.97	0.06	1.38	0.97-1.97	0.07
ACS	69/295	1.64	1.12-2.40	0.01	1.32	0.89-1.95	0.17	1.31	0.88-1.96	0.19

B. *Top CHIP driver genes vs no CHIP (reference)*

Characteristics	Cases / Total N	Model 1			Model 2			Model 3		
		OR	95%CI	*P*	aOR	95%CI	*P*	aOR	95%CI	*P*
**Severity CAD**										
No CAD	66/317	*Reference*								
Mild	44/317	2.16	1.42-3.28	3.38×10^{-4}	1.65	1.07-2.54	0.02	1.68	1.09-2.61	0.02
Moderate	29/317	1.44	0.90-2.30	0.13	1.03	0.63-1.68	0.91	1.06	0.65-1.73	0.83
Severe	178/317	1.54	1.14-2.09	5.0×10^{-3}	1.15	0.84-1.59	0.37	1.18	0.85-1.64	0.31
**Burden CAD**										
Non-obstructive CAD	73/251	*Reference*								
Single vessel	77/251	0.78	0.55-1.11	0.17	0.83	0.58-1.20	0.32	0.83	0.57-1.19	0.30
Multi-vessel	73/251	0.87	0.61-1.24	0.45	0.86	0.59-1.24	0.41	0.86	0.59-1.24	0.41
Left main	28/251	1.09	0.67-1.77	0.73	0.96	0.58-1.59	0.97	0.98	0.59-1.62	0.94

© 2025 Supriami K et al. *JAMA Network Open.*

Characteristics	Cases / Total N	OR	95%CI	P	aOR	95%CI	P	aOR	95%CI	P
Initial presentation										
No CAD	66/317	*Reference*								
Stable CAD	170/317	1.66	1.22-2.25	1.15x10^{-3}	1.17	0.85-1.62	0.33	1.20	0.86-1.67	0.28
ACS	81/317	1.51	1.07-2.15	0.02	1.28	0.89-1.83	0.19	1.33	0.92-1.92	0.13

Top driver genes included *DNMT3A*, *TET2*, *ASXL1*, and *JAK2*.

C. *Large clone (VAF ≥10%) vs no CHIP (reference)*

Characteristics	Cases / Total N	Model 1			Model 2			Model 3		
		**OR**	**95%CI**	***P***	**aOR**	**95%CI**	***P***	**aOR**	**95%CI**	***P***
**Severity CAD**										
No CAD	47/228	*Reference*								
Mild	25/228	1.72	1.03-2.88	0.04	1.13	0.66-1.94	0.65	1.17	0.68-2.01	0.57
Moderate	19/228	1.32	0.75-2.32	0.33	0.83	0.46-1.49	0.54	0.86	0.48-1.56	0.63
Severe	137/228	1.67	1.18-2.36	4.05x10^{-3}	1.17	0.81-1.69	0.41	1.21	0.82-1.76	0.33
**Burden CAD**										
Non-obstructive CAD	44/181	*Reference*								
Single vessel	54/181	0.91	0.60-1.39	0.67	1.05	0.68-1.64	0.82	1.07	0.69-1.66	0.77
Multi-vessel	64/181	1.27	0.84-1.91	0.26	1.34	0.87-2.05	0.19	1.32	0.85-2.03	0.22
Left main	19/181	1.23	0.69-2.19	0.49	1.07	0.59-1.95	0.83	1.10	0.60-2.01	0.76
**Initial presentation**										
No CAD	47/228	*Reference*								
Stable CAD	126/228	1.73	1.21-2.45	2.42x10^{-3}	1.09	0.75-1.58	0.66	1.12	0.76-1.65	0.56
ACS	55/228	1.44	0.96-2.18	0.08	1.17	0.76-1.79	0.47	1.22	0.79-1.89	0.37

Abbreviations: ACS, acute coronary syndromes; aOR, adjusted odds ratios; CAD, coronary artery disease.
Odd ratios (OR) with corresponding 95% confidence intervals (CI) in multivariable analyses were calculated using multinomial logistic regression models for categorical dependent variables. Model 1 was the crude, unadjusted analysis; model 2 included age at the time of coronary angiography, sex, and the first 4 principal components of genetic ancestry, as covariates; and model 3 was adjusted for age at the time of coronary angiography, sex, the first 4 principal components of genetic ancestry, hypertension, hypercholesterolemia, diabetes mellitus type 2, and current smoking. Multi-vessel was defined as a composite of 2-vessel or 3-vessel coronary lesions. ACS was defined as either ST-elevation myocardial infarction, non-ST-elevation myocardial infarction, or unstable angina. Patients with initial presentation of stable CAD or ACS but without any angiographic evidence of CAD was reclassified into the no CAD group (reference).

© 2025 Supriami K et al. *JAMA Network Open.*

eTable 16. Univariable and Multivariable-adjusted Cox Regression Models of Familial Hypercholesterolemia (FH) Variant, Coronary Artery Disease (CAD) Polygenic Risk Score (PRS), and Clonal Hematopoiesis of Indeterminate Potential (CHIP) Mutation

Outcomes	Cases / Total N	Model 1			Model 2			Model 3		
		HR	95%CI	*P*	aHR	95%CI	*P*	aHR	95%CI	*P*
**FH**										
Repeat angiogram										
Non-carrier	1,231/3,407	*Reference*								
Carrier	15/26	1.72	1.03-2.86	0.04	1.70	1.02-2.83	0.04	1.57	0.94-2.62	0.08
Revascularization										
Non-carrier	631/3,395	*Reference*								
Carrier	9/26	1.91	0.99-3.69	0.05	1.97	1.02-3.80	0.04	1.80	0.93-3.47	0.08
In-stent restenosis										
Non-carrier	282/3,381	*Reference*								
Carrier	4/25	1.87	0.70-5.02	0.22	1.78	0.66-4.80	0.25	1.61	0.60-4.34	0.34
Heart failure										
Non-carrier	231/1,551	*Reference*								
Carrier	0/7	NA	NA	NA	NA	NA	NA	NA	NA	NA
All-cause mortality										
Non-carrier	857/3,394	*Reference*								
Carrier	8/26	1.08	0.54-2.18	0.82	0.96	0.48-1.94	0.91	0.91	0.45-1.83	0.79
**CAD PRS**										
Repeat angiogram										
Low	103/380	*Reference*								
Intermediate	644/1,947	1.26	1.02-1.55	0.03	1.26	1.02-1.55	0.03	1.09	0.88-1.34	0.43
High	539/1,191	1.82	1.48-2.25	2.33×10^{-8}	1.79	1.45-2.22	5.92×10^{-8}	1.49	1.20-1.84	2.46×10^{-4}
Revascularization										
Low	50/380	*Reference*								
Intermediate	313/1,942	1.23	0.91-1.66	0.17	1.22	0.90-1.64	0.20	1.06	0.78-1.43	0.71
High	296/1,184	1.89	1.40-2.55	3.30×10^{-5}	1.85	1.37-2.50	5.76×10^{-5}	1.54	1.14-2.09	5.00×10^{-3}
In-stent restenosis										
Low	12/376	*Reference*								

© 2025 Supriami K et al. *JAMA Network Open.*

Outcomes	Cases / Total N	Model 1			Model 2			Model 3		
		HR	95%CI	*P*	aHR	95%CI	*P*	aHR	95%CI	*P*
Intermediate	122/1,936	1.97	1.09-3.57	0.03	1.98	1.09-3.58	0.02	1.71	0.94-3.11	0.08
High	152/1,179	3.98	2.21-7.16	$4.15x10^{-6}$	3.89	2.16-7.01	$6.19x10^{-6}$	3.17	1.75-5.73	$1.34x10^{-4}$
Heart failure										
Low	19/163	*Reference*								
Intermediate	140/902	1.38	0.85-2.22	0.19	1.39	0.86-2.25	0.18	1.27	0.78-2.06	0.34
High	81/538	1.23	0.75-2.03	0.42	1.34	0.81-2.22	0.25	1.15	0.69-1.92	0.59
All-cause mortality										
Low	88/377	*Reference*								
Intermediate	496/1,941	1.04	0.83-1.31	0.71	1.10	0.88-1.38	0.42	1.06	0.84-1.33	0.64
High	301/1,187	0.94	0.74-1.19	0.62	1.18	0.93-1.50	0.18	1.12	0.88-1.42	0.38
**CHIP**										
Repeat angiogram										
No CHIP	617/1,773	*Reference*								
Had CHIP	182/466	1.00	0.78-1.28	0.98	1.07	0.83-1.37	0.61	1.07	0.83-1.37	0.61
Revascularization										
No CHIP	294/1,765	*Reference*								
Had CHIP	97/466	1.27	0.94-1.72	0.12	1.18	0.86-1.62	0.29	1.15	0.84-1.58	0.37
In-stent restenosis										
No CHIP	114/1,760	*Reference*								
Had CHIP	37/463	0.99	0.59-1.64	0.97	1.00	0.60-1.70	0.98	0.98	0.58-1.66	0.95
Heart failure										
No CHIP	159/995	*Reference*								
Had CHIP	54/247	1.71	1.13-2.58	0.01	1.58	1.04-2.40	0.03	1.61	1.07-2.44	0.02
All-cause mortality										
No CHIP	429/1,767	*Reference*								
Had CHIP	172/465	2.41	1.99-2.92	$<2x10^{-16}$	1.78	1.47-2.16	$3.45x10^{-9}$	1.79	1.47-2.16	$3.15x10^{-9}$

Abbreviation: aHR, adjusted hazard ratios; CAD, coronary artery disease; CHIP, clonal hematopoiesis of indeterminate potential; FH, familial hypercholesterolemia; NA, not available; PRS, polygenic risk score.

Hazard ratios (HR) with corresponding 95% confidence intervals (CI) for the three genomic drivers of coronary artery disease, i.e. FH, CAD PRS, and CHIP, were calculated using Cox proportional hazards models. Model 1 was the crude, unadjusted analysis; model 2 included age at the time of coronary angiography, sex, and the first 4 principal components of genetic ancestry, as covariates; and model 3 was adjusted for age at the time of coronary angiography, sex, the first 4 principal components of genetic ancestry, hypertension, hypercholesterolemia, diabetes mellitus type 2, and current smoking. The grouping of CAD PRS was defined by the percentile distribution of CAD PRS, defined as low (bottom quintile), intermediate (middle three quintile), and high (top quintile). Revascularization was a composite outcome of percutaneous coronary

© 2025 Supriami K et al. *JAMA Network Open.*

intervention and coronary artery bypass graft. Heart failure was defined as chronic heart failure with any ejection fraction. Cox regression models were not run for heart failure by FH variant carries because there was no incident events occurred.

eTable 17. Age and Sex-matched Univariable and Multivariable-adjusted Cox Regression Models of Familial Hypercholesterolemia (FH) Variant

Outcomes	Cases / Total N	Model 1			Model 2			Model 3		
		HR	95%CI	*P*	aHR	95%CI	*P*	aHR	95%CI	*P*
Repeat angiogram										
Non-carrier	27/104	*Reference*								
Carrier	15/26	2.77	1.46-5.25	1.82×10^{-3}	2.83	1.46-5.50	2.18×10^{-3}	2.23	1.13-4.43	0.02
Revascularization										
Non-carrier	13/104	*Reference*								
Carrier	9/26	2.87	1.23-6.73	0.01	2.73	1.14-6.55	0.02	2.25	0.91-5.57	0.08
In-stent restenosis										
Non-carrier	4/103	*Reference*								
Carrier	4/25	3.95	0.99-15.81	0.05	5.12	1.19-22.09	0.03	4.22	0.90-19.73	0.07
Heart failure										
Non-carrier	7/49	*Reference*								
Carrier	0/7	NA	NA	NA	NA	NA	NA	NA	NA	NA
All-cause mortality										
Non-carrier	33/104	*Reference*								
Carrier	8/26	0.80	0.37-1.75	0.58	0.73	0.32-1.63	0.44	0.53	0.23-1.26	0.15

Abbreviation: aHR, adjusted hazard ratios; NA, not available.
Hazard ratios (HR) with corresponding 95% confidence intervals (CI) were calculated using Cox proportional hazards models. Model 1 was the crude, unadjusted analysis; model 2 included age at the time of coronary angiography, sex, and the first 4 principal components of genetic ancestry, as covariates; and model 3 was adjusted for age at the time of coronary angiography, sex, the first 4 principal components of genetic ancestry, hypertension, hypercholesterolemia, diabetes mellitus type 2, and current smoking. Revascularization was a composite outcome of percutaneous coronary intervention and coronary artery bypass graft. Heart failure was defined as chronic heart failure with any ejection fraction. Cox regression models were not run for heart failure because there was no incident events occurred in the FH carrier variant group.

© 2025 Supriami K et al. *JAMA Network Open.*

eTable 18. Multivariable-adjusted Cox Regression Models of Coronary Artery Disease (CAD) Polygenic Risk Score (PRS) Accounting for Baseline Disease Burden

Outcomes	Cases / Total N	aHR	95%CI	*P*
Repeat angiogram				
Low	103/380	*Reference*		
Intermediate	644/1,947	1.17	0.95-1.44	0.15
High	539/1,191	1.49	1.20-1.85	2.68×10^{-4}
Revascularization				
Low	50/380	*Reference*		
Intermediate	313/1,942	1.14	0.85-1.54	0.38
High	296/1,184	1.58	1.17-2.15	3.12×10^{-3}
In-stent restenosis				
Low	12/376	*Reference*		
Intermediate	122/1,936	1.84	1.02-3.34	0.04
High	152/1,179	3.26	1.80-5.91	9.72×10^{-5}
Heart failure				
Low	19/163	*Reference*		
Intermediate	140/902	1.37	0.85-2.22	0.20
High	81/538	1.30	0.78-2.17	0.31
All-cause mortality				
Low	88/377	*Reference*		
Intermediate	496/1,941	1.06	0.85-1.34	0.59
High	301/1,187	1.09	0.86-1.39	0.48

Abbreviation: aHR, adjusted hazard ratios.
Model was adjusted for age at the time of coronary angiography, sex, the first 4 principal components of genetic ancestry and Gensini score, as covariates.

© 2025 Supriami K et al. *JAMA Network Open.*

eTable 19. Univariable and Multivariable-adjusted Cox Regression Models of Coronary Artery Disease (CAD) Polygenic Risk Score (PRS) in the Replication Cohort (N=783)

**Outcomes**	**Cases / Total N**	**Model 1**			**Model 2**			**Model 3**		
		**HR**	**95%CI**	***P***	**aHR**	**95%CI**	***P***	**aHR**	**95%CI**	***P***
Repeat angiogram										
Low	23/87	*Reference*								
Intermediate	166/440	1.48	0.96-2.29	0.08	1.49	0.96-2.31	0.07	1.45	0.94-2.26	0.09
High	108/256	1.66	1.06-2.60	0.03	1.58	1.01-2.48	0.05	1.45	0.92-2.28	0.11
Revascularization										
Low	6/87	*Reference*								
Intermediate	82/440	2.78	1.21-6.36	0.02	3.63	1.32-9.95	0.01	2.63	1.14-6.06	0.02
High	53/256	2.89	1.24-6.73	0.01	3.83	1.38-10.62	0.01	2.58	1.10-6.01	0.03
In-stent restenosis										
Low	0/87	*Reference*								
Intermediate	33/440	NA	NA	NA	NA	NA	NA	NA	NA	NA
High	24/256	NA	NA	NA	NA	NA	NA	NA	NA	NA
Heart failure										
Low	6/40	*Reference*								
Intermediate	30/179	0.91	0.38-2.19	0.83	0.90	0.37-2.22	0.82	0.89	0.36-2.24	0.81
High	16/110	0.71	0.28-1.82	0.48	0.69	0.26-1.84	0.46	0.62	0.23-1.67	0.35
All-cause mortality										
Low	21/87	*Reference*								
Intermediate	142/437	1.25	0.79-1.98	0.34	1.17	0.74-1.86	0.51	1.12	0.71-1.79	0.62
High	87/253	1.21	0.75-1.95	0.44	1.29	0.80-2.09	0.30	1.16	0.72-1.89	0.54

Abbreviation: aHR, adjusted hazard ratios; CAD, coronary artery disease; NA, not available; PRS, polygenic risk score.
Hazard ratios (HR) with corresponding 95% confidence intervals (CI) were calculated using Cox proportional hazards models. Model 1 was the crude, unadjusted analysis; model 2 included age at the time of coronary angiography, sex, and the first 4 principal components of genetic ancestry, as covariates; and model 3 was adjusted for age at the time of coronary angiography, sex, the first 4 principal components of genetic ancestry, hypertension, hypercholesterolemia, diabetes mellitus type 2, and current smoking. The grouping of CAD PRS was defined by the percentile distribution of CAD PRS, defined as low (bottom quintile), intermediate (middle three quintile), and high (top quintile). Revascularization was a composite outcome of percutaneous coronary intervention and coronary artery bypass graft. Heart failure was defined as chronic heart failure with any ejection fraction. Cox regression models were not run for heart failure by FH variant carries because there was no incident events occurred. Not available (NA) was generated for the Cox regression analysis of in-stent restenosis because none of individuals in the low CAD PRS group, the reference group, experienced the outcome during follow-up.

© 2025 Supriami K et al. *JAMA Network Open.*

eTable 20. Cox Regression Models for All-cause Mortality by Clonal Hematopoiesis of Indeterminate Potential (CHIP) by Driver Genes and Clone Size

Outcomes	Cases / Total N	Model 1			Model 2			Model 3		
		HR	95%CI	*P*	aHR	95%CI	*P*	aHR	95%CI	*P*
**Non-*DNMT3A***										
Repeat angiogram										
No CHIP	617/1,773	*Reference*								
Non-*DNMT3A*	119/295	1.15	0.86-1.54	0.34	1.20	0.90-1.61	0.22	1.19	0.89-1.60	0.24
Revascularization										
No CHIP	294/1,765	*Reference*								
Non-*DNMT3A*	67/295	1.46	1.02-2.10	0.04	1.32	0.91-1.92	0.15	1.28	0.88-1.85	0.20
In-stent restenosis										
No CHIP	114/1,760	*Reference*								
Non-*DNMT3A*	27/293	1.33	0.76-2.32	0.32	1.34	0.76-2.37	0.32	1.28	0.72-2.28	0.40
Heart failure										
No CHIP	112/937	*Reference*								
Non-*DNMT3A*	26/140	1.84	1.11-3.04	0.02	1.73	1.04-2.86	0.03	1.75	1.06-2.89	0.03
All-cause mortality										
No CHIP	429/1,767	*Reference*								
Non-*DNMT3A*	123/294	2.74	2.20-3.40	$<2x10^{-16}$	1.94	1.56-2.42	$4.21x10^{-9}$	1.93	1.55-2.41	$4.93x10^{-9}$
**Top driver genes**										
Repeat angiogram										
No CHIP	617/1,773	*Reference*								
Top driver genes	118/317	0.93	0.69-1.25	0.63	0.99	0.74-1.33	0.95	1.01	0.75-1.35	0.96
Revascularization										
No CHIP	294/1,765	*Reference*								
Top driver genes	60/317	1.21	0.84-1.73	0.30	1.12	0.78-1.62	0.54	1.11	0.77-1.61	0.58
In-stent restenosis										
No CHIP	114/1,760	*Reference*								
Top driver genes	23/315	0.93	0.50-1.73	0.81	0.94	0.50-1.76	0.84	0.93	0.50-1.75	0.83
Heart failure										
No CHIP	112/937	*Reference*								

© 2025 Supriami K et al. *JAMA Network Open.*

Outcomes	Cases / Total N	Model 1			Model 2			Model 3		
		HR	95%CI	*P*	aHR	95%CI	*P*	aHR	95%CI	*P*
Top driver genes	30/172	1.28	0.75-2.19	0.37	1.18	0.69-2.02	0.55	1.21	0.71-2.07	0.49
All-cause mortality										
No CHIP	429/1,767	*Reference*								
Top driver genes	109/316	2.16	1.72-2.71	3.48×10^{-11}	1.63	1.31-2.04	1.64×10^{-5}	1.64	1.31-2.06	1.43×10^{-5}
**Clone size**										
Repeat angiogram										
No CHIP	617/1,773	*Reference*								
VAF ≥10%	76/228	0.80	0.56-1.14	0.22	0.86	0.60-1.24	0.42	0.86	0.60-1.24	0.42
Revascularization										
No CHIP	294/1,765	*Reference*								
VAF ≥10%	43/228	1.05	0.66-1.65	0.84	0.98	0.61-1.55	0.92	0.95	0.60-1.52	0.83
In-stent restenosis										
No CHIP	114/1,760	*Reference*								
VAF ≥10%	16/226	0.58	0.24-1.40	0.23	0.60	0.24-1.50	0.28	0.60	0.24-1.51	0.28
Heart failure										
No CHIP	112/937	*Reference*								
VAF ≥10%	18/112	1.74	0.98-3.07	0.06	1.59	0.89-2.85	0.12	1.66	0.93-2.97	0.09
All-cause mortality										
No CHIP	429/1,767									
VAF ≥10%	96/227	2.97	2.34-3.78	$<2 \times 10^{-16}$	2.00	1.57-2.56	2.43×10^{-8}	2.01	1.57-2.57	2.75×10^{-8}

Abbreviation: aHR, adjusted hazard ratios; CHIP, clonal hematopoiesis of indeterminate potential; VAF, variant allele fraction.
Hazard ratios (HR) with corresponding 95% confidence intervals (CI) were calculated using time-dependent Cox regression models. Model 1 was the crude, unadjusted analysis; model 2 included age at the time of coronary angiography, sex, and the first 4 principal components of genetic ancestry, as covariates; and model 3 was adjusted for age at the time of coronary angiography, sex, the first 4 principal components of genetic ancestry, hypertension, hypercholesterolemia, diabetes mellitus type 2, and current smoking. Any top driver genes are defined as a presence of either *DNMT3A*, *TET2*, *AXSL1*, or *JAK2* as the driver gene of the patient.

© 2025 Supriami K et al. *JAMA Network Open.*

eTable 21. Cox Regression Models for All-cause Mortality by CAD PRS and Clonal Hematopoiesis of Indeterminate Potential Restricted on Individuals Sequenced either Before or Within a Maximum of 1 Year before Their First Coronary Angiography

All-cause mortality	Cases / Total N	Model 1			Model 2			Model 3		
		HR	95%CI	*P*	aHR	95%CI	*P*	aHR	95%CI	*P*
**CAD PRS**										
Low	36/170	*Reference*								
Intermediate	167/815	0.96	0.67-1.38	0.82	0.98	0.68-1.41	0.92	0.94	0.65-1.36	0.75
High	86/453	0.80	0.54-1.18	0.26	0.96	0.64-1.42	0.82	0.90	0.60-1.33	0.59
**CHIP**										
No CHIP	118/600	*Reference*								
Had CHIP	48/148	2.01	1.43-2.81	4.85×10^{-5}	1.54	1.09-2.18	0.01	1.54	1.08-2.19	0.02

Abbreviation: aHR, adjusted hazard ratios; CAD, coronary artery disease; CHIP, clonal hematopoiesis of indeterminate potential; PRS, polygenic risk score.
Hazard ratios (HR) with corresponding 95% confidence intervals (CI) were calculated using Cox proportional hazards models and time-dependent Cox regression models for CHIP. Model 1 was the crude, unadjusted analysis; model 2 included age at the time of coronary angiography, sex, and the first 4 principal components of genetic ancestry, as covariates; and model 3 was adjusted for age at the time of coronary angiography, sex, the first 4 principal components of genetic ancestry, hypertension, hypercholesterolemia, diabetes mellitus type 2, and current smoking. The grouping of CAD PRS was defined by the percentile distribution of CAD PRS, defined as low (bottom quintile), intermediate (middle three quintile), and high (top quintile). Cox regression models were not run for FH variant carries because there was no incident events occurred after the restriction.

© 2025 Supriami K et al. *JAMA Network Open.*

eReferences

1. Patel AP, Wang M, Ruan Y, et al. A multi-ancestry polygenic risk score improves risk prediction for coronary artery disease. Nat Med. 2023;29(7):1793-1803. doi:10.1038/s41591-023-02429-x
2. Hao, L., Kraft, P., Berriz, G.F. et al. Development of a clinical polygenic risk score assay and reporting workflow. Nat Med 28, 1006–1013 (2022). https://doi.org/10.1038/s41591-022-01767-6
3. Patel AP, Fahed AC. Pragmatic Approach to Applying Polygenic Risk Scores to Diverse Populations. Current Protocols. 2023;3(11):e911. doi:https://doi.org/10.1002/cpz1.911
4. Cho SMJ, Lee H, Koyama S, Zou RS, Schuermans A, Ganesh S, Hornsby W, Honigberg MC, Natarajan P. Cumulative Diastolic Blood Pressure Burden in Normal Systolic Blood Pressure and Cardiovascular Disease. Hypertension. 2024 Feb;81(2):273-281.
5. Cho SMJ, Koyama S, Honigberg MC et al. Genetic, sociodemographic, lifestyle, and clinical risk factors of recurrent coronary artery disease events: a population-based cohort study. Eur Heart J 2023;44:3456-3465.

© 2025 Supriami K et al. *JAMA Network Open.*
