# Supplementary material for: Genomic Drivers of Coronary Artery Disease and Risk of Future Outcomes After Coronary Angiography
Source: JAMA Netw Open. 2025 Jan 21;8(1):e2455368. doi: 10.1001/jamanetworkopen.2024.55368 (PMC11751748; doi:10.1001/jamanetworkopen.2024.55368)
Supplement: Supplement 2. — Data Sharing Statement [file jamanetwopen-e2455368-s002.pdf]

Data Sharing Statement

Supriami. Genomic Drivers of Coronary Artery Disease and Risk of Future Outcomes After Coronary Angiography. *JAMA Netw Open*. Published January 21, 2025. doi:10.1001/jamanetworkopen.2024.55368

Data
Data available: No

Additional Information
Explanation for why data not available: Individual patient data on the whole-exome sequencing (WES) and other related analyses, together with individual-level phenotypes and outcomes are available through restricted access on the Mass General Brigham (MGB) Biobank portal.
